# *In vivo* Regeneration of Ganglion Cells for Vision Restoration in Mammalian Retinas

**DOI:** 10.3389/fcell.2021.755544

**Published:** 2021-10-04

**Authors:** Dongchang Xiao, Kangxin Jin, Suo Qiu, Qiannan Lei, Wanjing Huang, Haiqiao Chen, Jing Su, Qiang Xu, Zihui Xu, Bin Gou, Xiaoxiu Tie, Feng Liu, Sheng Liu, Yizhi Liu, Mengqing Xiang

**Affiliations:** ^1^State Key Laboratory of Ophthalmology, Zhongshan Ophthalmic Center, Sun Yat-sen University, Guangzhou, China; ^2^Guangdong Provincial Key Laboratory of Brain Function and Disease, Zhongshan School of Medicine, Sun Yat-sen University, Guangzhou, China

**Keywords:** retinal ganglion cell, cell reprogramming, retinal ganglion cell regeneration, glaucoma, transcription factor

## Abstract

Glaucoma and other optic neuropathies affect millions of people worldwide, ultimately causing progressive and irreversible degeneration of retinal ganglion cells (RGCs) and blindness. Previous research into cell replacement therapy of these neurodegenerative diseases has been stalled due to the incapability for grafted RGCs to integrate into the retina and project properly along the long visual pathway. *In vivo* RGC regeneration would be a promising alternative approach but mammalian retinas lack regenerative capacity. It therefore has long been a great challenge to regenerate functional and properly projecting RGCs for vision restoration in mammals. Here we show that the transcription factors (TFs) Math5 and Brn3b together are able to reprogram mature mouse Müller glia (MG) into RGCs. The reprogrammed RGCs extend long axons that make appropriate intra-retinal and extra-retinal projections through the entire visual pathway to innervate both image-forming and non-image-forming brain targets. They exhibit typical neuronal electrophysiological properties and improve visual responses in RGC loss mouse models. Together, our data provide evidence that mammalian MG can be reprogrammed by defined TFs to achieve *in vivo* regeneration of functional RGCs as well as a promising new therapeutic approach to restore vision to patients with glaucoma and other optic neuropathies.

## Introduction

Glaucoma is a neurodegenerative disorder characterized by progressive and irreversible degeneration of retinal ganglion cells (RGCs) and the optic nerve, and is the second leading cause of blindness worldwide (Quigley and Broman, 2006; Quigley, 2011). Despite its discovery almost a century and half ago (Grewe, 1986), there is currently still no cure for glaucoma and other optic neuropathies. RGCs project their axons along a long visual pathway through the optic nerve, optic chiasm and optic tract to connect to their appropriate central targets in the brain (McLaughlin and O’Leary, 2005; Petros et al., 2008; Crair and Mason, 2016; Herrera et al., 2019). They are the only output neurons in the retina that transmit visual signals from the retina to the brain, and as such, are critical for sight. During development, RGCs are guided by a variety of neurotrophic factors and guidance cues to successfully navigate the complex visual pathway (Oster et al., 2004; McLaughlin and O’Leary, 2005; Petros et al., 2008; Crair and Mason, 2016; Herrera et al., 2019). However, adult RGCs lose their ability to respond to the guidance cues perhaps due to the downregulation in expression of the corresponding receptors and signaling molecules and other intrinsic changes (Chen et al., 1995; Crair and Mason, 2016; Benowitz et al., 2017). Because of the numerous guidance barriers needed to overcome by transplanted RGCs to reach the brain targets (Crair and Mason, 2016; Benowitz et al., 2017), cell transplantation therapies using donor RGCs or iPSC-derived RGCs to treat RGC degenerative diseases have been unsuccessful even in mammalian animal models. For instance, intra-retinally directing grafted RGCs to extend axons toward the optic disk has proven to be a major challenge since grafted RGCs usually grow axons in random directions (Kador et al., 2013). The use of a scaffold may improve transplantation therapies but major progress has yet to be made to realize its potential (Kador et al., 2013, 2014; Li et al., 2017).

Apart from cell transplantation treatments, *in vivo* RGC regeneration would be an ideal therapy but mammalian retinas are thought to lack regenerative capacity. In spite of this, the Müller glia (MG) have been shown to serve as retinal stem cells to repair injured retinas in cold-blood vertebrates such as zebrafish (Bernardos et al., 2007; Goldman, 2014; Lenkowski and Raymond, 2014). Similarly, mammalian MG display stem cell-like/late retinal progenitor features, e.g., having a molecular signature similar to that of the late retinal progenitors (Roesch et al., 2008; Jadhav et al., 2009; Dvoriantchikova et al., 2019), exhibiting limited proliferative and neurogenic capacity in damaged retinas (Ooto et al., 2004; Karl et al., 2008; Ueki et al., 2015; Wilken and Reh, 2016; Jorstad et al., 2017), and transdifferentiating into rods by a combination of β-catenin and transcription factors (TFs) (Yao et al., 2018). Thus, a fundamental question that remains to be answered is whether MG can be induced to efficiently regenerate functional and properly projecting RGCs for vision restoration in mammals. We sought to harness the stem cell-like property of MG to regenerate RGCs *in vivo* by TF-directed reprogramming. During murine retinogenesis, the bHLH TF Math5/Atoh7 is transiently expressed in a subset of retinal progenitors and required for conferring them with the competence of RGC generation (Brown et al., 2001; Wang et al., 2001; Yang et al., 2003; Xiang, 2013). Previously, we and others have demonstrated the expression of the POU-domain transcription factor Brn3b/Pou4f2 in RGC precursors and its crucial function in RGC specification and differentiation (Xiang et al., 1993; Erkman et al., 1996; Gan et al., 1996; Xiang, 1998, 2013; Qiu et al., 2008). We thus investigated the ability of the Math5 and Brn3b TF combination to reprogram adult mouse MG into RGCs. Remarkably, we were able to show that without stimulating proliferation, Math5 together with Brn3b reprogrammed mature mouse MG into RGCs while either alone had no or limited capacity. The reprogrammed RGCs were functional, extended long axons through the entire visual pathway to innervate both image-forming and non-image-forming targets in the brain, and improved visual responses in two RGC loss mouse models: *Brn3b* null mutant mice and mice with the optic nerve crushed (ONC).

## Results

### Reprogramming of Müller Glia Into Retinal Ganglion Cells by Math5 and Brn3b

**FIGURE 1:**
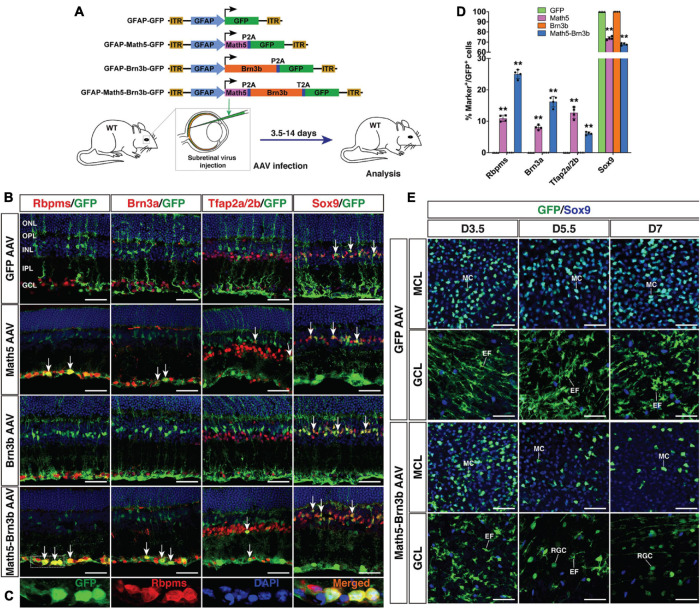
Generation of RGCs by TF-mediated reprogramming of adult mouse MG. **(A)** Schematic of the AAV constructs and infection procedure to generate RGCs in adult wild-type (WT) mice. **(B,C)** Two weeks after infection with GFAP-GFP, GFAP-Math5-GFP, GFAP-Brn3b-GFP or GFAP-Math5-Brn3b-GFP AAVs, sections from infected retinas were double-immunolabeled with the indicated antibodies and counterstained with nuclear DAPI. Arrows point to representative co-labeled cells. Shown in **(C)** are higher magnification single-plane confocal images of the outlined region in **(B)**. **(D)** Quantitation of GFP+ cells that become immunoreactive for Rbpms, Brn3a, Tfap2a/2b or Sox9 in retinas infected with GFAP-GFP, GFAP-Math5-GFP, GFAP-Brn3b-GFP, or GFAP-Math5-Brn3b-GFP AAVs. Data are presented as mean ± SD (*n* = 4). Asterisks indicate significance in two-way ANOVA test with Bonferroni’s correction: **p < 0.0001. **(E)** At 3.5 (D3.5), 5.5 and 7 days after viral infection, flat-mounts of adult mouse retinas infected with GFAP-GFP or GFAP-Math5-Brn3b-GFP AAVs were immunostained for both GFP and Sox9. The confocal images are focused on the Müller cell layer (MCL) or ganglion cell layer (GCL). EF, MG endfoot; GCL, ganglion cell layer; INL, inner nuclear layer; IPL, inner plexiform layer; MC, Müller cell; MCL, Müller cell layer; ONL, outer nuclear layer; OPL, outer plexiform layer; RGC, retinal ganglion cell. Scale bars = 40 μm **(B,E)**.

**FIGURE 2:**
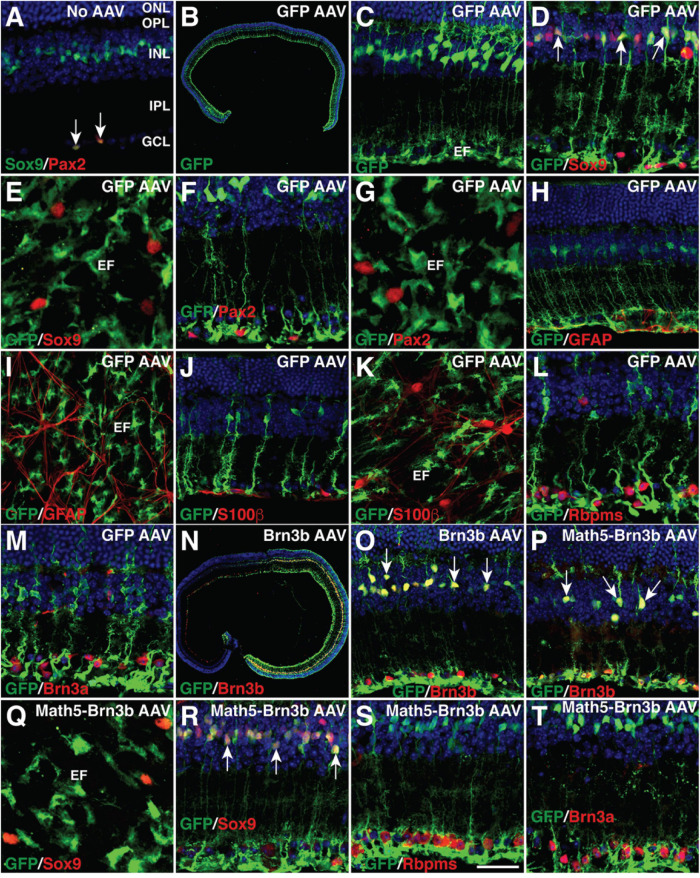
MG-specificity of the GFAP promoter in adult mouse retinas. **(A)** Sections from retinas without AAV infection were double-immunolabeled with antibodies against Sox9 and Pax2 and counterstained with nuclear DAPI. Arrows point to co-labeled cells. Sox9 and Pax2 are completely co-localized in astrocytes within the GCL. **(B–M)** Two weeks after infection with GFAP-GFP AAVs, sections **(B–D,F,H,J,L,M)** or flat-mounts **(E,G,I,K)** from infected retinas were immunolabeled with the indicated antibodies. Sections were also counterstained with nuclear DAPI. Arrows point to representative co-labeled cells. The GFP+ cells are not immunoreactive for Sox9, Pax2, GFAP, or S100β in the GCL **(D–K)**, nor are they immunoreactive for Rbpms or Brn3a **(L,M)**. **(N,O)** Two weeks after infection with GFAP-Brn3b-GFP AAVs, sections from infected retinas were immunolabeled with antibodies against GFP and Brn3b and counterstained with nuclear DAPI. Arrows point to representative co-labeled cells. **(P–T)** At 3.5 **(Q–T)** and 5.5 **(P)** days after infection with GFAP-Math5-Brn3b-GFP AAVs, sections **(P,R–T)** or flat-mount **(Q)** from infected retinas were immunolabeled with the indicated antibodies. Sections were also counterstained with nuclear DAPI. Arrows point to representative co-labeled cells. At day 3.5, the GFP+ cells are not immunoreactive for Sox9 in the GCL **(Q,R)**, nor are they immunoreactive for Rbpms or Brn3a **(S,T)**. EF, MG endfoot; GCL, ganglion cell layer; INL, inner nuclear layer; IPL, inner plexiform layer; ONL, outer nuclear layer; OPL, outer plexiform layer. Scale bar: 640 μm **(B)**, 457 μm **(N)**, 40 μm **(C,H–K,O,P)**, 30 μm **(A,D,F,L,M,R–T)**, 20 μm **(E,G,Q)**.

To regenerate RGCs *in vivo*, MG-specific expression of TFs was achieved by a GFAP promoter in the adeno-associated viruses (AAVs, serotype 9 or ShH10) injected subretinally into the adult mouse eyes (Figure 1A). Two weeks after infection with the GFAP-GFP AAVs, numerous MG located in the inner nuclear layer (INL) of the retina were seen to express GFP and display a typical Müller cell morphology with processes spanning both the inner and outer retinal layers (Figures 2B,C). The GFP+ cells were immunoreactive only for the MG marker Sox9 in the INL but not for the astrocyte markers Pax2, Sox9, GFAP, or S100β in the ganglion cell layer (GCL), nor were they immunoreactive for RGC markers Rbpms or Brn3a in the GCL (Figures 2A,D–M). Thus, the GFAP promoter we used in this study was highly specific to MG without driving reporter expression in astrocytes and RGCs. Consistent with this, we found that GFAP-Brn3b-GFP and GFAP-Math5-Brn3b-GFP AAVs mediated Brn3b expression in MG only (Figures 1A, 2N–P). Moreover, at 3.5 days following infection, GFAP-Math5-Brn3b-GFP AAVs did not drive GFP expression in either Sox9-immunoreactive astrocytes or RGCs immunoreactive for Rbpms and Brn3a (Figures 2Q–T).

By 2 weeks after viral infection, compared to MG infected with control GFAP-GFP AAVs, we found that many MG infected with GFAP-Math5-Brn3b-GFP AAVs changed their morphology, lost MG processes and migrated into the GCL (Figure 1B). They were immunoreactive for RGC markers Rbpms and Brn3a or for the amacrine cell marker Tfap2a/2b, but not for the Müller cell marker Sox9 (Figures 1B,C). Quantification of immunoreactive cells revealed that the Math5 and Brn3b combination reprogrammed infected MG into 25.0% Rbpms+ RGCs, 16.2% Brn3a+ RGCs, and 6.1% Tfap2a/2b+ amacrine cells with 67.9% remained as Sox9+ Müller cells, whereas MG infected with control GFP AAVs remained as 100% Sox9+ Müller cells (Figures 1B,D). Single Brn3b factor did not exert any reprogramming effect although single Math5 TF converted infected MG into 10.9% Rbpms+ RGCs, 8.0% Brn3a+ RGCs, and 12.7% Tfap2a/2b+ amacrine cells (Figures 1B,D). These results indicate that Math5 together with Brn3b are able to reprogram mature MG into RGCs whereas either TF alone has no or weaker capacity. The reprogrammed RGCs included some cells that expressed melanopsin, Eomes or peripherin, which are protein markers for ipRGCs (Mao et al., 2014), some that expressed Foxp2 and Brn3c, which are markers for F- and F-midi^ON^ RGCs, respectively (Rousso et al., 2016), as well as those expressing Satb2, a maker for three RGC subtypes: On-Off DSGC, Off DSGC, and Off-sustained RGC (Dhande et al., 2019; Supplementary Figure 1). Thus, MG appear to be converted into different RGC subtypes by Math5 and Brn3b.

**FIGURE 3:**
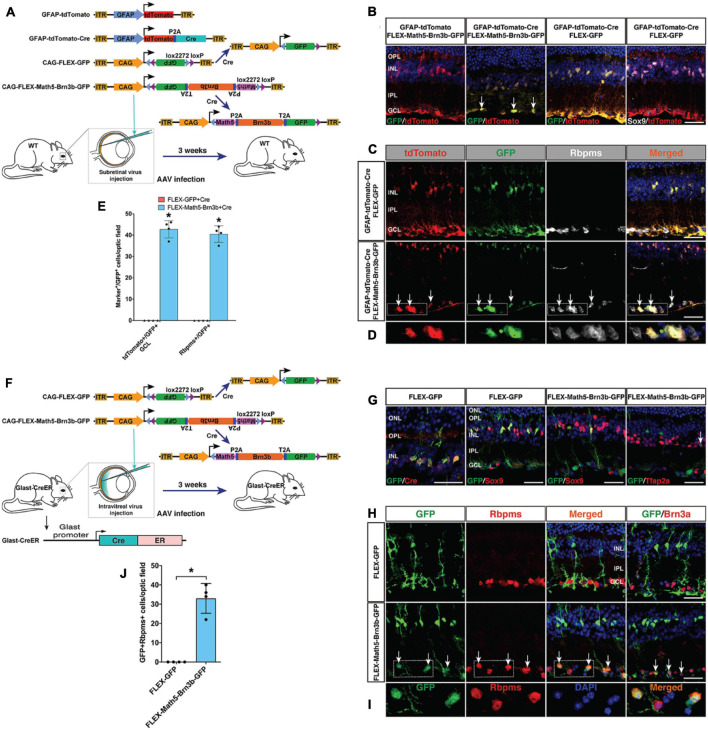
Lineage tracing analysis of MG-derived RGCs. **(A)** Schematic of the AAV constructs and infection procedure as well as the resulting FLEX AAV constructs in the presence of Cre. **(B)** Three weeks after injection of the indicated AAVs, sections from infected retinas were double-immunolabeled with the indicated antibodies and counterstained with DAPI. Arrows point to representative double-positive cells located within the GCL. **(C,D)** Three weeks after injection of the indicated AAVs, sections from infected retinas were triple-immunolabeled with the indicated antibodies and counterstained with DAPI. Arrows point to representative triple-positive cells located within the GCL. Shown in **(D)** are higher magnification single-plane confocal images of the outlined region in **(C)**. **(E)** Quantitation of tdTomato+/GFP+ cells in the GCL as well as Rbpms+/GFP+ cells. Data are presented as mean ± SD (*n* = 4). Asterisks indicate significance in unpaired two-tailed Student’s *t*-test: *p < 0.0001. **(F)** Schematic of the FLEX AAV constructs, resulting FLEX AAV constructs in the presence of Cre, infection procedure, and the Glast-CreER mice. **(G)** Three weeks after injection of the indicated AAVs, sections from infected retinas were double-immunolabeled with the indicated antibodies and counterstained with DAPI. The arrow points to a double-positive cell located within the INL. **(H,I)** Three weeks after injection of the indicated AAVs, sections from infected retinas were double-immunolabeled with the indicated antibodies and counterstained with DAPI. Arrows point to representative double-positive cells located within the GCL. Shown in **(I)** are higher magnification single-plane confocal images of the outlined region in **(H)**. **(J)** Quantitation of GFP+/Rbpms+ double-positive RGCs. Data are presented as mean ± SD (*n* = 4). Asterisks indicate significance in unpaired two-tailed Student’s *t*-test: *p < 0.0005. GCL, ganglion cell layer; INL, inner nuclear layer; IPL, inner plexiform layer; OPL, outer plexiform layer. Scale bars = 30 μm **(B,C,G,H)**.

To confirm that RGCs were indeed reprogrammed from MG by Math5 and Brn3b, we performed cell lineage-tracing analysis using the FLEX Cre-Switch system (Figure 3A). In wild-type retinas infected with both CAG-FLEX-Math5-Brn3b-GFP and GFAP-tdTomato-Cre AAVs, there were many Rbpms+ RGCs that were also immunoreactive for tdTomato and GFP; whereas essentially all GFP+/tdTomato+ cells were restricted to the INL and immunoreactive for Sox9 in retinas infected with both CAG-FLEX-GFP and GFAP-tdTomato-Cre AAVs (Figures 3B–E). Moreover, GFP+/tdTomato+ cells were not seen in retinas infected with both CAG-FLEX-Math5-Brn3b-GFP and GFAP-tdTomato AAVs (Figure 3B). These results suggest that the newly generated RGCs arose from tdTomato-marked MG in cell lineage. To more stringently trace reprogrammed RGCs, we further utilized the tamoxifen-inducible Glast-CreER transgenic line (Heng et al., 2019; Figure 3F). These mice were infected with CAG-FLEX-Math5-Brn3b-GFP or CAG-FLEX-GFP AAVs, immediately followed by 4 daily tamoxifen administrations, and analyzed at 21 days post virus-injection. In retinas infected with control CAG-FLEX-GFP AAVs, GFP+ cells were found only in the INL, which were co-labeled for Cre and Sox9 (Figure 3G), confirming the MG-specificity of Cre expression in the Glast-CreER animals. By contrast, in retinas infected with CAG-FLEX-Math5-Brn3b-GFP AAVs, there were not only GFP+ cells that were immunoreactive for Sox9 in the INL but also many GFP+ cells that were positive for Rbpms or Brn3a in the GCL and some GFP+ cells positive for Tfap2a (Figures 3G–I). In fact, we found GFP+/Rbpms+ double-positive RGCs only in retinas infected with CAG-FLEX-Math5-Brn3b-GFP AAVs but not in those infected with CAG-FLEX-GFP AAVs (Figures 3H–J), demonstrating that the reprogrammed RGCs were derived from MG and that *Math5* and *Brn3b* DNA sequences do not affect the AAV tropism or expression specificity.

### Temporal, Morphological, and Molecular Changes During Müller Glia Transdifferentiation

To determine the onset time during which Math5/Brn3b-mediated MG transdifferentiation occurs, we immunolabeled retinal flat-mounts of 3.5, 5.5, and 7 days post-infection with antibodies against GFP and Sox9 (Figure 1E). Infection by GFAP-Math5-Brn3b-GFP AAVs resulted in numerous GFP+/Sox9+ double-positive MG in the Müller cell layer at day 3.5, which were decreased by days 5.5 and 7 (Figure 1E). The AAVs did not drive reporter gene expression in RGCs by day 3.5 as evidenced by the lack of GFP+ RGCs within the GCL, but some round GFP+ RGCs began to emerge in the GCL by day 5.5 and they became more numerous by day 7 (Figure 1E). In contrast, the number of MG infected by control GFAP-GFP AAVs did not display any obvious change from day 3.5 to 7, and even by day 7, there were no GFP+ RGCs visible in the GCL of retinas infected with control AAVs (Figure 1E). The dynamic cellular changes induced by Math5 and Brn3b thus suggest that during the MG reprogramming process, the transdifferentiation events take place 3.5 days after viral infection.

**FIGURE 4:**
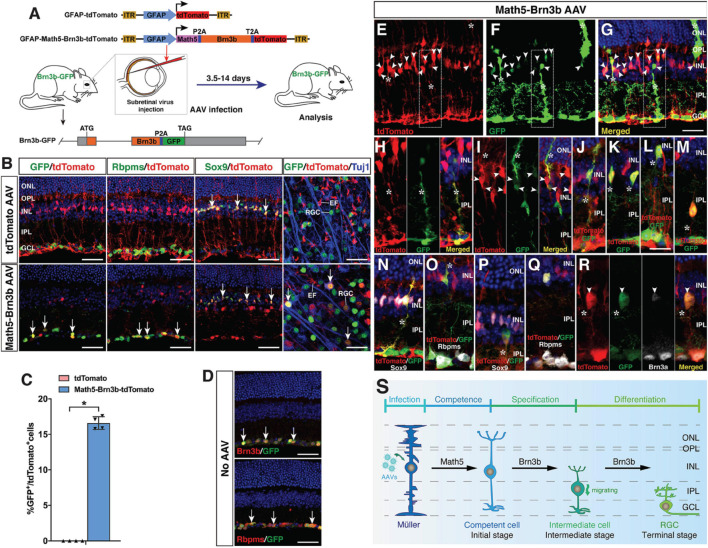
Initial, intermediate and terminal stages of MG transdifferentiation induced by Math5 and Brn3b. **(A)** Schematic of the AAV constructs and infection procedure to generate RGCs in Brn3b-GFP reporter mice. **(B)** Two weeks after infection with the indicated AAVs, sections or flat-mounts from infected retinas were double-immunolabeled with the indicated antibodies. Sections were also counterstained with DAPI. Note that fluorescent staining for Rbpms and Sox9 is in manually assigned false green color. Arrows point to representative co-labeled cells. **(C)** Quantitation of tdTomato+ cells that become immunoreactive for GFP 3 weeks following infection with GFAP-Math5-Brn3b-tdTomato AAVs. Data are presented as mean ± SD (*n* = 4). Asterisks indicate significance in unpaired two-tailed Student’s *t*-test: *p < 0.0001. **(D)** Sections from retinas of Brn3b-GFP reporter mice without AAV infection were double-immunolabeled with the indicated antibodies and counterstained with DAPI. Arrows point to representative co-labeled cells. **(E–R)** At day 4–6 post-infection with GFAP-Math5-Brn3b-tdTomato AAVs, sections from infected retinas were double- or triple-immunolabeled with the indicated antibodies and counterstained with DAPI. Shown in **(H)** is a higher magnification view of the region outlined in **(E–G)**. **(I,J)** show more representative images of the initial-stage cells that co-express tdTomato and GFP. **(K–M)** show intermediate-stage cells located at the inner edge of the INL and in the IPL with the MG morphology lost. Note that the MG-like cells at the initial stage were labeled by tdTomato, GFP and Sox9 but not by Rbpms **(N,O)**, whereas the bigger cells at the intermediate stage were labeled by tdTomato, GFP, Rbpms, and Brn3a but not by Sox9 **(P–R)**. Arrowheads point to representative co-labeled cells. Asterisks indicate co-labeled MG-like and other cellular processes. **(S)** Proposed changes and mechanism during the MG-to-RGC transdifferentiation process. AAV-mediated Math5 expression may confer RGC competence to MG, on which Brn3b may subsequently act to drive RGC specification and differentiation. During reprogramming, MG undergo morphological and molecular changes including losing MG processes and marker expression while gaining RGC morphology and marker expression. EF, MG endfoot; GCL, ganglion cell layer; INL, inner nuclear layer; IPL, inner plexiform layer; ONL, outer nuclear layer; OPL, outer plexiform layer; RGC, retinal ganglion cell. Scale bars = 40 μm **(B,D)**, 30 μm **(E–G)**, 20 μm **(H–R)**.

To further monitor the MG reprogramming process, we generated a Brn3b-GFP reporter mouse line in which GFP was simultaneously expressed with Brn3b to specifically mark both immature and mature RGCs (Figures 4A,D). Thus, most MG-derived RGCs in this line would be labeled by the GFP reporter whenever the expression of the endogenous *Brn3b* was turned on. Indeed, by 2–3 weeks post-infection, within the GCL of retinas infected with GFAP-Math5-Brn3b-tdTomato AAVs, the majority of tdTomato-positive cells were also immunoreactive for GFP as well as Rbpms, whereas in control retinas, tdTomato-positive cells remained immunoreactive only for Sox9 but negative for either GFP or Rbpms (Figure 4B). Quantification of immunoreactive cells showed that there were 16.6% of GFP+ cells in all tdTomato+ cells within retinas infected with GFAP-Math5-Brn3b-tdTomato AAVs (Figure 4C).

As described above, the terminal stage of MG transdifferentiation induced by Math5 and Brn3b is represented by the differentiated RGCs located in the GCL. Based on the determined onset time of MG reprogramming (Figure 1E), we next searched for the initial and intermediate states of MG transdifferentiation in the Brn3b-GFP reporter mouse line at 4–6 days following infection with GFAP-Math5-Brn3b-tdTomato AAVs. As expected, in this time window, many MG-like cells within the INL were seen to express both tdTomato and various levels of GFP, representing the initial stage of MG transdifferentiation (Figures 4E–J). By GFP labeling, some of the cells with high GFP expression exhibited a MG-like morphology and co-expressed Sox9 but not the mature RGC marker Rbpms (Figures 4E–J,N,O), whereas in retinas without AAV infection, GFP, as a RGC-specific marker expressed from the endogenous *Brn3b* gene locus, was never found in the MG (Figure 4D). We have shown previously that Brn3b is the earliest known RGC marker with an onset expression in RGC precursors (Gan et al., 1996; Xiang, 1998). So the cells at the initial transdifferentiation stage may represent those competent for RGC generation (Figure 4S). In addition, we found GFP+/tdTomato+ double-positive cells located at the inner edge of the INL or in the inner plexiform layer, with larger cell bodies and short processes, representing a migratory intermediate state between the initial and terminal stages of MG transdifferentiation (Figures 4K–M,P–R). These cells gradually lost the MG morphology, did not express Sox9 but instead expressed more mature RGC markers Rbpms and Brn3a (Figures 4P–R), indicating that their fate was already specified/determined as RGCs (Figure 4S). Therefore, Math5 and Brn3b rather quickly induce temporal, morphological, and molecular changes in MG to direct them toward the RGC differentiation pathway during reprogramming.

To further confirm the existence of intermediate stages during the reprogramming process, we enriched GFP+ cells in wild-type retinas 5.5 days post-infection with GFAP-Math5-Brn3b-GFP AAVs, and subjected them to scRNA-seq analysis. Among 23238 sequenced cells, 1214 cells were GFP+. Because the cells at intermediate stages are expected to be GFP+, we subjected these GFP+ cells to pseudotime trajectory analysis, which yielded several states along which the expression of the RGC marker genes *Tubb3* (*Tuj1*), *Gap43* and *Sncg* gradually increases while the expression of the MG marker genes *Sox2* and *Sox9* progressively decreases (Supplementary Figures 2A,B). Moreover, there are many cells that express both *Sox9* and *Rbpms, Gap43* or *Syt4* (synaptotagmin IV) (Supplementary Figures 2C–F), indicating the presence of intermediate cells expressing both MG and RGC marker genes. UMAP visualization of all 23238 sequenced cells revealed a group of related cell populations expressing *Rbpms*, *Sox2* or both (Supplementary Figure 2H), which was re-analyzed by UMAP. This analysis clearly demonstrates trajectories reflecting the transition process from MG to intermediate stage RGCs where *Sox2*, *Sox9*, and *Slc1a3* (*Glast*) are mostly expressed at high levels in MG but at low levels in intermediate stage RGCs, while the opposite is true for *Rbpms*, *Sox4* and *Stmn2* (Supplementary Figures 2H–M). Other more mature RGC marker genes *Sncg*, *Ebf1*, and *Irx3* display a later onset expression during the process (Supplementary Figures 2N–P). Notably, *Sox4* has recently been identified as a marker for developmentally nascent RGCs (Wu et al., 2021). Thus, MG-to-RGC reprogramming undergoes intermediate states and corresponding molecular changes.

To determine if proliferation is required for MG-to-RGC reprogramming, we investigated whether MG proliferation was stimulated by Math5 and Brn3b. The retinas were labeled by EdU injection at day 2.5 or 4.5 following infection with GFAP-Math5-Brn3b-GFP AAVs, and harvested 1 or 4.5 days later; or labeled by EdU at day 7, 14, 21, and 28 and harvested at day 30 (Supplementary Figure 3A). In all cases, essentially no EdU-positive MG or other cells were observed (Supplementary Figure 3), suggesting that Math5 together with Brn3b are able to reprogram mature MG into RGCs without triggering their proliferation.

### Proper Projection of Müller Glia-Derived Retinal Ganglion Cells in the Visual Pathway

**FIGURE 5:**
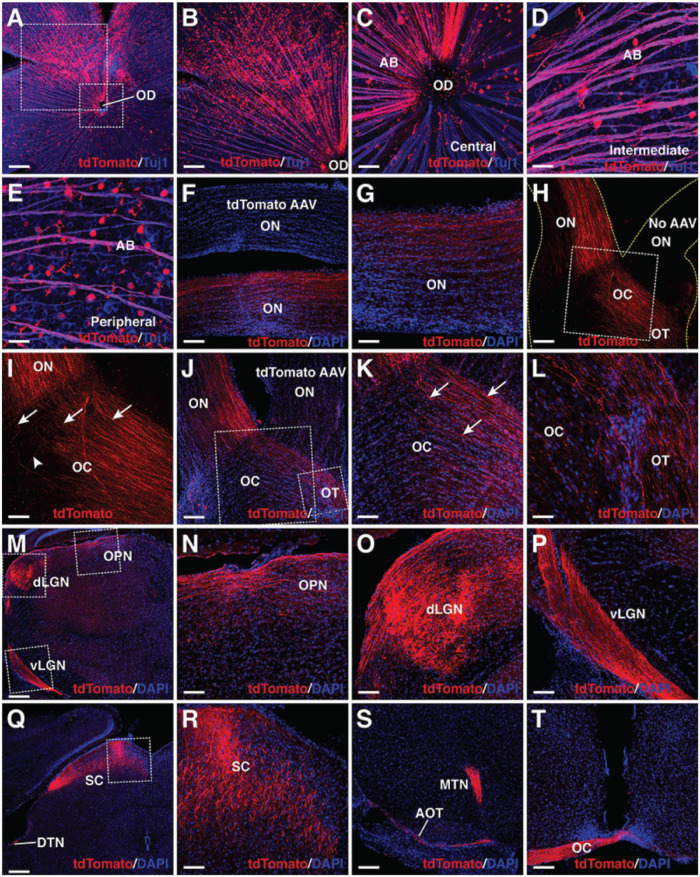
MG-derived RGCs recapitulate the visual projection pathway of endogenous RGCs. **(A–E)** Flat-mounts of wild-type adult mouse retinas treated with GFAP-Math5-Brn3b-tdTomato AAVs were double-immunolabeled with anti-tdTomato and anti-Tuj1 antibodies. The two areas outlined by the large and small squares in **(A)** are shown at a higher magnification in **(B,C)**, respectively. Shown in **(C–E)** are representative images from the central, intermediate and peripheral retinas, respectively. **(F,G)** The optic nerves of mice treated with GFAP-Math5-Brn3b-tdTomato or GFAP-tdTomato (tdTomato AAV) AAVs were immunolabeled with an anti-tdTomato antibody and counterstained with DAPI. **(H,I)** Mono-ocular treatment of adult mice with GFAP-Math5-Brn3b-tdTomato AAVs revealed that tdTomato-immunoreactive axons projected predominantly into the contralateral optic tract. The yellow dashed lines in **(H)** outline the optic nerve and chiasm regions. The optic nerve from the uninjected eye (No AAV) is indicated. Shown in **(I)** is a higher magnification view of the region outlined in **(H)**. Arrows in **(I)** point to axons crossing the midline of the optic chiasm while the arrowhead indicates a non-crossing axon. **(J–L)** The optic nerves, optic chiasms and optic tracts of mice treated with GFAP-Math5-Brn3b-tdTomato AAVs in one eye and GFAP-tdTomato AAVs in the other were immunolabeled with an anti-tdTomato antibody and counterstained with DAPI. The two areas outlined by the large and small squares in **(J)** are shown at a higher magnification in **(K)** and **(L)**, respectively. Arrows in **(K)** point to axons crossing the midline of the optic chiasm. **(M–T)** Brain areas innervated by MG-derived RGCs in mice treated with GFAP-Math5-Brn3b-tdTomato AAVs. Shown in **(N–P)** are higher magnification views of the corresponding outlined regions in **(M)** and shown in **(R)** is a higher magnification view of the region outlined in **(Q)**. AB, axon bundle; AOT, accessory optic tract; dLGN, dorsal lateral geniculate nucleus; DTN, dorsal terminal nucleus; EF, MG endfoot; MTN, medial terminal nucleus; OC, optic chiasm; OD, optic disk; ON, optic nerve; OPN, olivary pretectal nucleus; OT, optic tract; RGC, retinal ganglion cell; SC, superior colliculus; vLGN, ventral lateral geniculate nucleus. Scale bars = 320 μm **(A,M,Q)**, 160 μm **(B,F,H,J,S,T)**, 80 μm **(G,I,K,N,O,P,R)**, 40 μm **(C–E,L)**.

Endogenous RGC axons project along a stereotypic visual pathway to connect to their appropriate central targets in the brain (Petros et al., 2008; Crair and Mason, 2016; Herrera et al., 2019). We investigated whether *in vivo* regenerated RGCs had the ability to overcome the guidance obstacles to make correct projection along the same pathway. At 3–4 weeks following infection of the adult retina by GFAP-Math5-Brn3b-tdTomato AAVs, numerous tdTomato-immunoreactive RGCs were observed on the vitreous surface of the retina, which extended axons that were fasciculated into many thick axon bundles immunoreactive for Tuj1 (Figures 5A–E). Remarkably, these bundles extended all the way from the periphery through intermediate and central retinal areas to enter the optic disk (Figures 5A–E). Once exiting the optic disk, these RGC axons continued to navigate through the optic nerve (Figures 5F,G). The great majority of them crossed over the midline of the optic chiasm to continue their projection in the contralateral optic tract while a small number continued their projection in the ipsilateral optic tract (Figures 5H–L). The predominant contralateral axon projection was confirmed by treating one eye with GFAP-Math5-Brn3b-tdTomato AAVs and the other with control GFAP-tdTomato AAVs or no AAVs, which unambiguously showed that the great majority of axons from MG-derived RGCs crossed the midline of the optic chiasm (Figures 5H,J). In the brain, there were plenty of tdTomato+ axons from MG-derived RGCs that reached and innervated various central targets responsible for both image-forming and non-image forming vision: the lateral geniculate nucleus, superior colliculus, olivary pretectal nucleus, terminal nucleus, accessory optic tract, and the above-mentioned optic chiasm (Figures 5M–T). Therefore, MG-derived RGCs are able to extend long axons capable of navigating the entire visual pathway to innervate proper central targets. By contrast, adult eyes without treatment or treated with control GFAP-tdTomato AAVs did not project tdTomato+ axons into the optic nerve (Figures 5F,H,J and Supplementary Figures 5B–E).

We further confirmed by rabies-virus *trans-*synaptic tracing that MG-derived RGCs innervate neurons of the lateral geniculate nucleus that project to the primary visual cortex (V1). In this experiment, RGCs were converted from MG by GFAP-Math5-Brn3b-tdTomato AAVs and the ΔG-RABV-GFP rabies viruses were injected into the V1 area of visual cortex. As expected, we observed a fraction of RGCs that were immunoreactive for both tdTomato and GFP (Supplementary Figure 4). In addition, we investigated the onset and temporal progression of MG-derived RGC projections. By 3.5 days post-infection with the GFAP-Math5-Brn3b-tdTomato AAVs, no tdTomato+ axons were seen in the optic nerve (Supplementary Figure 5F), in agreement with the observation that there were no RGCs reprogrammed from MG by this time (Figure 1E). Half a day later, however, tdTomato+ axons entered the optic nerve and traveled a small distance (Supplementary Figure 5G). By day 5.5, there were some tdTomato+ axons that traversed the entire optic nerve to reach the optic chiasm but few appeared to cross over it (Supplementary Figures 5H,I). By day 7, however, there were abundant tdTomato+ axons that reached the optic chiasm and crossed its midline (Supplementary Figures 5J,K). Thus, the onset and progression patterns of MG-derived RGC axons closely follow the temporal window of MG transdifferentiation (Figure 1E).

### Retinal Ganglion Cell Regeneration in Young and Aged *Brn3b* Null Mutant Mice

**FIGURE 6:**
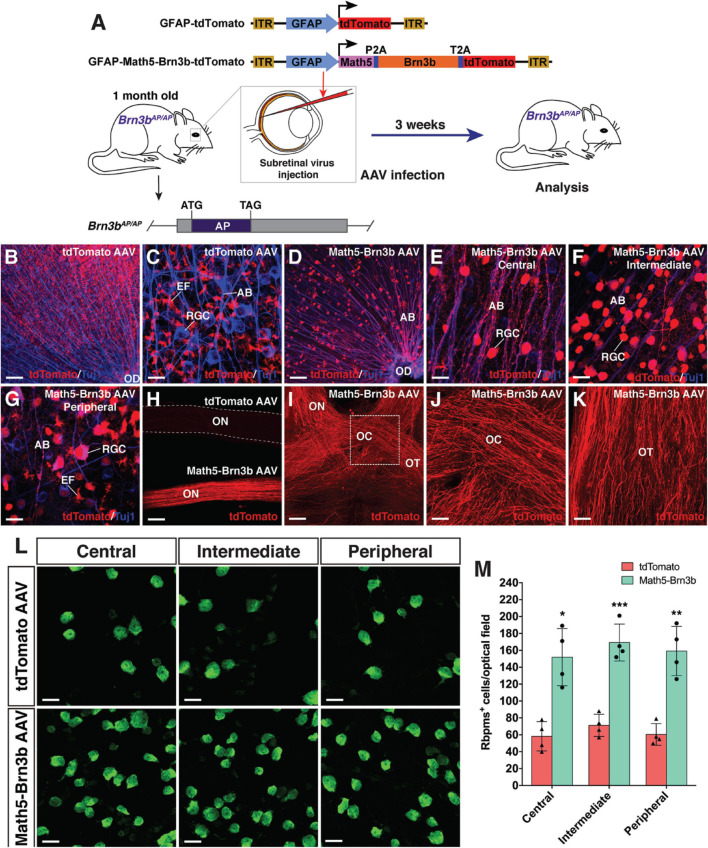
RGC regeneration in *Brn3b*^*AP/AP*^ mice. **(A)** Schematic of the AAV constructs and infection procedure to regenerate RGCs in *Brn3b*^*AP/AP*^ mice at 1 month of age. **(B,C)** Flat-mounts of *Brn3b*^*AP/AP*^ retinas treated with GFAP-tdTomato AAVs were double-immunolabeled with anti-tdTomato and anti-Tuj1 antibodies. **(D–G)** Flat-mounts of *Brn3b*^*AP/AP*^ retinas treated with GFAP-Math5-Brn3b-tdTomato AAVs were double-immunolabeled with anti-tdTomato and anti-Tuj1 antibodies. Shown in **(E–G)** are representative images from the central, intermediate and peripheral retinas, respectively. **(H)** The optic nerves from *Brn3b*^*AP/AP*^ mice treated with GFAP-Math5-Brn3b-tdTomato AAVs were immunoreactive for tdTomato whereas those from *Brn3b*^*AP/AP*^ mice treated with GFAP-tdTomato AAVs were not. **(I–K)** The optic nerves, optic chiasms and optic tracts from *Brn3b*^*AP/AP*^ mice treated with GFAP-Math5-Brn3b-tdTomato AAVs were immunoreactive for tdTomato. Shown in **(J)** is a higher magnification view of the region outlined in **(I)**. **(L)** Flat-mounts of central, intermediate and peripheral *Brn3b*^*AP/AP*^ retinas treated with GFAP-tdTomato or GFAP-Math5-Brn3b-tdTomato AAVs were immunostained with an anti-Rbpms antibody. **(M)** Quantification of Rbpms+ cells in central, intermediate and peripheral *Brn3b*^*AP/AP*^ retinas treated with GFAP-tdTomato or GFAP-Math5-Brn3b-tdTomato AAVs. Data are presented as mean ± SD (*n* = 4). Asterisks indicate significance in unpaired two-tailed Student’s *t*-test: *p < 0.005, **p < 0.001, ***p < 0.0005. AB, axon bundle; EF, MG endfoot; OC, optic chiasm; OD, optic disk; ON, optic nerve; OT, optic tract; RGC, retinal ganglion cell. Scale bars = 114 μm **(I)**, 80 μm **(B,D,H)**, 40 μm **(J,K)**, 20 μm **(C,E–G,L)**.

To evaluate the functionality of regenerated RGCs, we attempted to reprogram MG into RGCs in *Brn3b*^*AP/AP*^ knockout mutant mice (1 month old) where 70–80% of RGCs are lost (Gan et al., 1996, 1999; Figure 6). On the vitreous surface of mutant retinas infected with GFAP-tdTomato AAVs, except for numerous tdTomato-positive MG endfeet, there were no RGCs and axon bundles labeled by tdTomato (Figures 6B,C). By contrast, in mutant retinas infected with GFAP-Math5-Brn3b-tdTomato AAVs, many tdTomato-immunoreactive RGCs were present and these regenerated RGCs extended numerous tdTomato-positive axon bundles that exhibited proper projection to the optic disk (Figures 6D–G). Moreover, these nerve fibers navigated all the way through the optic nerve, optic chiasm and optic tract whereas no tdTomato-positive axons were seen in the control optic nerve (Figures 6H–K). Overall, there was two to three-fold increase of RGCs in the central, intermediate and peripheral regions in retinas infected with GFAP-Math5-Brn3b-tdTomato AAVs (Figures 6L,M and Supplementary Figure 6A). Thus, similar to in wild-type retinas, RGCs can be efficiently reprogrammed from MG in *Brn3b*^*AP/AP*^ null mutant retinas as well. Consistent with this, transmission electron microscopy revealed an increase of axon density as well as axons with thick myelin sheath in optic nerves of *Brn3b*^*AP/AP*^ mice treated with GFAP-Math5-Brn3b-tdTomato AAVs, compared to those treated with GFAP-tdTomato AAVs (Supplementary Figure 6B).

We investigated age-dependency of this TF-mediated *in vivo* RGC regeneration by performing similar experiments in 8-month-old *Brn3b*^*AP/AP*^ mice (Supplementary Figure 7). In these animals treated with GFAP-Math5-Brn3b-tdTomato AAVs, RGCs were also increased by approximately two-fold and the regenerated RGCs extended axons all the way from the retina to the optic tract and displayed predominant contralateral projection at the optic chiasm (Supplementary Figure 7). Therefore, MG can be reprogrammed to regenerate RGCs even in aged animals.

### Regenerated Retinal Ganglion Cells Improve Visual Function in Mouse Models of Retinal Ganglion Cell Loss

**FIGURE 7:**
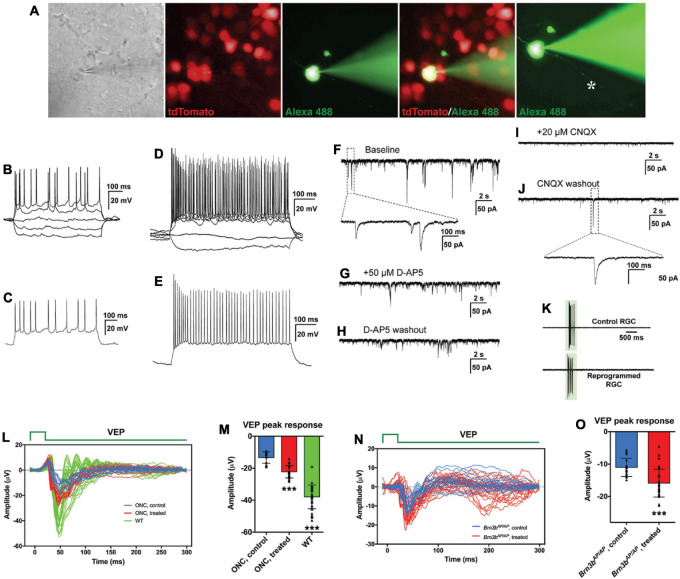
MG-derived RGCs improve visual function of RGC loss mouse models. **(A–J)** Patch-clamp recordings of reprogrammed RGCs in mouse retinas at 3 weeks after treatment with GFAP-Math5-Brn3b-tdTomato AAVs. **(A)** An MG-derived RGC marked by tdTomato (red) was chosen for patch-clamp recording. Alexa Fluor 488 hydrazide was used to confirm the recorded cell and visualize the axon (indicated by the asterisk). **(B,D)** Current-clamp recordings revealed action potential responses of the MG-derived RGCs under current injection. **(C,E)** Action potentials were induced after depolarization of the patched cells. **(F–J)** Representative traces of spontaneous excitatory postsynaptic currents (sEPSCs) of an MG-derived RGC. Voltage clamp was performed and responses were recorded under baseline condition **(F)**, D-AP5 application **(G)**, D-AP5 washout **(H)**, CNQX application **(I)**, and CNQX washout **(J)**. **(K)** Light-evoked spikes of control and reprogrammed RGCs were obtained with current-clamp recording. The duration of light stimulus is indicated by the green rectangle. **(L,M)** Visual evoked responses (VEPs) to a flash light in the visual cortex of ONC mouse models in right eyes (WT, without optic nerve crush, *n* = 7) and in left eyes (optic nerve crushed) treated with GFAP-Math5-Brn3b-GFP AAVs (ONC, treated, *n* = 4) or GFAP-GFP AAVs (ONC, control, *n* = 3). VEP recordings were performed at 6 weeks after AAV treatment. Shown in **(L)** are responses from all trials and five trials were performed for each eye. Shown in **(M)** are amplitudes of the VEP response peaks for control, treated and WT eye groups. Points represent single trials. Data are presented as mean ± SD. Asterisks indicate significance in one-way ANOVA test with Bonferroni’s correction: ***p < 0.0001. **(N,O)** VEPs to a flash light in the visual cortex of *Brn3b*^*AP/AP*^ mice treated with GFAP-Math5-Brn3b-tdTomato AAVs (*Brn3b*^*AP/AP*^, treated, *n* = 6 eyes) or GFAP-tdTomato AAVs (*Brn3b*^*AP/AP*^, control, *n* = 4 eyes). VEP recordings were performed at 4 weeks after AAV treatment. Shown in **(N)** are responses from all trials. Shown in **(O)** are amplitudes of the positive VEP response peaks for control and treated eye groups. Points represent single trials. Data are presented as mean ± SD. Asterisks indicate significance in unpaired two-tailed Student’s *t*-test: ***p < 0.0001.

To determine whether we reprogrammed MG into functional RGC neurons, we infected adult wild-type and Brn3b-GFP reporter mouse retinas with GFAP-Math5-Brn3b-tdTomato AAVs. Three weeks after viral infection, patch-clamp recording was carried out for the MG-derived RGCs that were located in the GCL and displayed the tdTomato red fluorescence (Figure 7A). We included the green fluorophore Alexa Fluor 488 in the internal solution to confirm that the recorded cells were also labeled by tdTomato and found that the well-filled cells extended a long visible axon (Figure 7A). The great majority of recorded neurons (17 out of 18) had multiple action potential responses (Figures 7B–E). To investigate the synaptic mechanism of the reprogrammed RGCs, we recorded spontaneous postsynaptic currents (sPSCs) (Figures 7F–J). We found that D-AP5, an NMDA receptor antagonist, slightly decreased the amplitude of the sPSCs (Figures 7G,H), while CNQX, a competitive AMPA/kainate receptor antagonist, blocked almost all the events (Figure 7I), and the effect could be abolished by washing out the drug (Figure 7J). Moreover, patch-clamp recording showed that the light responses of reprogrammed RGCs were similar to those of unlabeled endogenous RGCs (Figure 7K). These results thus suggest that RGCs reprogrammed from MG are able to differentiate into mature functional neurons, develop ionotropic glutamate receptors to receive excitatory inputs, and integrate into the retinal neural circuits.

Given the apparently proper projection of the MG-derived RGCs along the visual pathway, we tested whether they were able to transmit light responses to the primary visual cortex *in vivo*. Six weeks after AAV treatment, visual evoked potentials (VEPs) to a flash light in the primary visual cortex were recorded from well-characterized RGC loss mouse models with the optic nerve crushed (ONC) (Supplementary Figures 8F,G). From ONC eyes infected with GFAP-GFP AAVs (control), the light stimulus elicited much smaller VEP responses compared to those from normal wild-type eyes. Infection of the ONC eyes with GFAP-Math5-Brn3b-GFP AAVs (treated) triggered obviously stronger VEP responses than the control treatment (Figure 7L). Quantification showed that the amplitudes of the VEP response peaks from the treated eyes were increased by ∼ 67% compared to those from the control eyes (Figure 7M). In agreement, in treated ONC animals, many labeled axons extended beyond the injury site of the optic nerve and reached brain targets including the lateral geniculate nucleus and superior colliculus, whereas hardly any axons extended beyond the injury site in control ONC mice (Supplementary Figure 8). Additionally, 4 weeks after AAV treatment, we recorded VEPs from *Brn3b*^*AP/AP*^ mice infected with GFAP-Math5-Brn3b-tdTomato AAVs (treated) or GFAP-tdTomato AAVs (control). In agreement with the loss of 70–80% RGCs in *Brn3b* knockout animals (Gan et al., 1996), the control mutant eyes gave only small VEP responses (Figure 7N). Again, the light stimulus triggered stronger VEP responses from the treated eyes, with the response amplitudes increased by ∼ 44% compared to control eyes (Figure 7O), consistent with the observed increase of RGCs in the treated retina (Figure 6). However, when VEP recordings were performed at 2 weeks following AAV infection, we did not observe significant difference in VEP response amplitudes between the treated and control groups in either ONC models or *Brn3b* mutant mice (Supplementary Figure 9), suggesting that it may take more than 2 weeks for the regenerated RGCs to form functional visual neural circuits.

## Discussion

### Mammalian Müller Glia Can Be Reprogrammed Into Retinal Ganglion Cells by Defined Transcription Factors

Despite the fact that mammalian MG have little regenerative capacity, our present study demonstrates that mature mouse MG can be reprogrammed by Math5 and Brn3b TFs into functional RGCs that make proper axon projections and have appropriate electrophysiological properties. The result is remarkable and one may wonder whether the reprogrammed RGCs might actually result from infected extant endogenous RGCs that non-specifically express reporters. However, multiple lines of evidence indicate that this is extremely unlikely: (1) We have shown that the GFAP promoter used in this work is completely specific to MG. We did not observe any GFP+ RGCs owing to infection by control GFAP-GFP AAVs; (2) Infection of MG by GFAP-GFP or GFAP-Brn3b-GFP AAVs alone did not produce any GFP+ RGCs. Robust number of GFP+ RGCs appeared only when GFAP-Math5-Brn3b-GFP AAVs were used for infection; (3) By 3.5 days post-infection of retinas with GFAP-Math5-Brn3b-GFP AAVs, many MG but no RGCs were GFP+; only after 5.5 days post-infection were GFP+ RGCs seen, consistent with the time-window of RGC reprogramming and the appearance of intermediate cells (Figures 1, 4). Thus, GFAP-Math5-Brn3b-GFP AAVs have no ability to drive GFP reporter expression in existing RGCs and the resulting GFP+ RGCs must be derived from infected MG; (4) By genetic labeling and single cell transcriptomics, we observed the initial and intermediate states of MG transdifferentiation mediated by Math5 and Brn3b, which were accompanied with temporal, morphological and molecular changes characteristic of RGC reprogramming (Figure 4 and Supplementary Figure 2); (5) Our lineage tracing experiments demonstrated that the reprogrammed GFP+ RGCs were all derived from MG; (6) tdTomato-positive axons were observed only in optic nerves extended from eyes treated with GFAP-Math5-Brn3b-tdTomato AAVs but not in optic nerves from eyes treated with control GFAP-tdTomato AAVs; and (7) Infection with GFAP-Math5-Brn3b-tdTomato AAVs increased RGC density by up to three-fold even in the absence of ∼70–80% of endogenous RGCs in the *Brn3b*^*AP/AP*^ retina (Figure 6 and Supplementary Figure 7). This indicates that the newly generated RGCs are reprogrammed rather than AAV-infected extant RGCs because it would be impossible to observe increased RGC density even if all extant RGCs were infected by AAVs and expressed the tdTomato reporter. Therefore, these lines of evidence strongly argue that the RGCs increased by infection with AAVs expressing both Math5 and Brn3b were not extant endogenous RGCs but reprogrammed from MG.

Another possibility to explain our data is that RGCs might be reprogrammed from astrocytes residing in the GCL rather than from MG. However, we can essentially rule out this possibility because following infection by GFAP-GFP AAVs, there were no GFP+ cells present in the GCL that co-localized with either Pax2, Sox9, GFAP, or S100β which are astrocyte markers. Moreover, as shown in this study (Figure 2) and by others (Stanke et al., 2010), astrocytes in adult mouse retinas represent a very small cell population, which can hardly explain the numerous reprogrammed RGCs present in the GCL in the absence of cell proliferation.

In this work, we have provided evidence that mature MG are able to be reprogrammed by defined TFs to generate functional RGCs even without activation by injury or proliferation-stimulants. This is in contrast to previous observation of neurogenesis and rod generation by murine MG, which does require prior MG activation (Ooto et al., 2004; Karl et al., 2008; Ueki et al., 2015; Jorstad et al., 2017; Yao et al., 2018), suggesting a possible cell type-specificity of this requirement. In addition, we have shown that the RGC reprogramming efficiency from MG by TFs is similar in both young and aged *Brn3b* null mutant mice, making it feasible to treat not only young but also aged patients with glaucoma and other optic neuropathies by this regeneration strategy.

### *In vivo* Reprogrammed Retinal Ganglion Cells Are Functional and Make Proper Projection in the Visual Pathway

Our study shows that the *in vivo* reprogrammed RGCs migrate into the GCL and make proper intra-retinal and extra-retinal projections through the entire visual pathway to innervate both image-forming and non-image-forming targets in the brain. These results implicate that even in the adult organism, the mammalian visual system may still maintain a relatively intact and permissive environment for regenerated RGC axons to outgrow and navigate to appropriate brain targets, and that unlike postnatal endogenous RGCs which lose their ability to extend (Chen et al., 1995), *in vivo* regenerated RGCs retain the ability to project axons along the visual pathway, similar to embryonically born RGCs (Chen et al., 1995). Although the obstacles to regenerate mammalian RGCs with appropriate central projections once appeared to be insurmountable due to countless guidance barriers present along the long visual pathway (Crair and Mason, 2016), evidence is accumulating that RGC axons regenerated *in vivo* or derived from transplanted donor cells do have the ability to properly navigate the adult visual pathway. For instance, in mice, a combinatorial treatment of *Pten* deletion and injection with Zymosan and CPT-cAMP stimulated regeneration of RGC axons that traversed the entire visual pathway to reach correct brain target zones including the lateral geniculate nucleus, superior colliculus, olivary pretectal nucleus, and terminal nucleus (de Lima et al., 2012). Similarly, combining neural activity with mTOR activation led to regeneration of RGC axons that projected to the correct brain visual targets (Lim et al., 2016). A very recent study reports that RGCs converted from MG by *Ptbp1* knockdown project properly to the central targets (Zhou et al., 2020). Apart from regenerated RGC axons, axons extended from transplanted early postnatal RGCs also had a limited capability of correct intra-retinal projection and navigation across the optic chiasm to reach the dorsal and ventral lateral geniculate nucleus and superior colliculus (Venugopalan et al., 2016). Therefore, adult mammalian visual system most likely still maintains a permissive environment to allow for normal or properly regenerated RGCs to make correct projections and establish accurate neural circuit connections.

During visual system development, RGCs newly generated from RPCs express a series of attractive and repulsive guidance molecules and TFs such as Ephrins, Neuropilin 1, Slits, DCC, DSCAM, Vax1, and Zic2, to ensure their accurate axon pathfinding through the visual pathway to innervate the correct brain processing centers (Oster et al., 2004; McLaughlin and O’Leary, 2005; Erskine and Herrera, 2007; Petros et al., 2008; Crair and Mason, 2016; Herrera et al., 2019). Because MG are similar to late retinal progenitors (Roesch et al., 2008; Jadhav et al., 2009; Dvoriantchikova et al., 2019), it is likely that the RGCs newly reprogrammed from MG by Math5 and Brn3b express a similar repertoire of guidance cues such that they are also able to readily navigate the complex visual pathway. Adult RGCs may downregulate expression of these critical guidance molecules and undergo unfavorable intrinsic changes while ES/iPSC-derived RGCs may fail to express the complete repertoire of guidance receptors and signaling molecules due to the lack of proper developmental milieu. Thus, when transplanted, adult RGCs and ES/iPSC-derived RGCs may have great difficulty to navigate the visual pathway due to their inability to respond to the guidance cues. Given the many desirable feats of regenerated RGCs never achieved by transplanted retinal stem cells or *in vitro* differentiated RGCs, *in vivo* regeneration of RGCs by MG through directed reprogramming provides a promising new therapeutic approach to restore vision to patients with glaucoma and related optic neuropathies.

The RGCs reprogrammed from MG had action potential responses and excitatory postsynaptic potentials typical of functional neurons and responded to lights. These electrophysiological properties combined with their ability to project to and innervate proper brain visual targets foretold their integration in the visual system and the formation of effective neural circuits. Indeed, after treatment with AAVs expressing both Math5 and Brn3b for 4–6 weeks, we observed a significant increase of VEP responses in both *Brn3b*^*AP/AP*^ and ONC mice, indicating that *in vivo* regenerated RGCs are able to improve visual function in RGC loss mouse models. However, no significant difference was seen in VEP responses after treating these model animals for only 2 weeks, suggesting that just like embryonic RGCs, it may take weeks for the reprogrammed RGCs to differentiate and mature properly, navigate the lengthy visual pathway, and make appropriate central connections.

### Mechanism of Müller Glia-to-Retinal Ganglion Cell Reprogramming by Transcription Factors

In this study, we used Math5 and Brn3b, two TFs critical for the specification and differentiation of RGCs during development, to efficiently reprogram MG into RGCs in the adult retina, suggesting that MG reprogramming events may largely recapitulate the molecular events that occur in development. Previously, we and others have shown that during retinogenesis, Math5 is required for conferring RPCs with the competence of RGC generation and its overexpression promotes RGC differentiation by RPCs (Brown et al., 2001; Liu et al., 2001; Wang et al., 2001; Yang et al., 2003). Moreover, Brn3b acts downstream of Math5 and plays an essential role in RGC specification and differentiation (Xiang et al., 1993; Erkman et al., 1996; Gan et al., 1996; Xiang, 1998; Liu et al., 2001; Qiu et al., 2008). In particular, we showed that overexpression of Brn3b alone was able to promote RGC differentiation in early RPCs but unable to do so in postnatal mouse RPCs (Liu et al., 2000; Qiu et al., 2008), suggesting that postnatal RPCs lose the competence of generating RGCs. This loss of competence most likely results from the demonstrated downregulation of Math5 expression in postnatal retinas (Brown et al., 1998). Given that mouse MG have a molecular signature similar to that of late postnatal RPCs (Roesch et al., 2008; Jadhav et al., 2009; Dvoriantchikova et al., 2019), it is no wonder that MG do not have any competence of RGC generation. However, in our reprogramming scheme, with the replenishment of Math5 by AAV-mediated expression, MG may gain the competence of RGC generation. Overexpressed Brn3b may then act on the competent MG to promote RGC generation and differentiation (Figure 4S), hence making it possible to regenerate RGCs *in vivo* from mammalian MG.

The fact that Math5 alone was able to convert 10.9% MG into RGCs suggests that it indeed has the ability to make MG competent for RGC generation. But because Math5 is not a RGC determination factor (Yang et al., 2003), it also reprogrammed MG into 12.7% amacrine cells. The addition of Brn3b increased the fraction of reprogrammed RGCs but reduced that of reprogrammed amacrine cells, in agreement with our previous finding that Brn3b promotes the RGC fate while inhibiting amacrine cell development (Qiu et al., 2008). Apart from Math5 and Brn3b, there are other TFs such as Isl1 and Sox factors involved in RGC specification and differentiation (Mu et al., 2008; Pan et al., 2008; Jiang et al., 2013; Xiang, 2013; Wu et al., 2015; Chang et al., 2017; Kuwajima et al., 2017). It will be interesting to determine whether these TFs also have an activity in reprogramming MG into RGCs.

In summary, to search for RGC regeneration strategies for potential therapy and vision restoration in damaged and diseased retinas, we made a remarkable discovery that the mammalian MG can be reprogrammed by developmentally pertinent TFs Math5 and Brn3 into functional RGCs. The MG-to-RGC transdifferentiation occurs in the absence of cell proliferation. The reprogrammed RGCs extend long axons that traverse the optic nerve and project predominantly into the contralateral optic tract through the optic chiasm to innervate both image-forming and non-image-forming brain targets such as lateral geniculate nucleus and superior colliculus. They display typical neuronal electrophysiological features and improve vision in RGC loss mouse models. With the great difficulties RGC transplantation therapy is currently facing, our work of successful *in vivo* RGC regeneration may provide a powerful approach leading to therapeutics for glaucoma, optic dystrophy, diabetic retinopathy and other optic neuropathies.

## Materials and Methods

### Animals

All experiments on mice were performed according to the Institutional Animal Care and Use Committee (IACUC) standards, and approved by Sun Yat-sen University and Zhongshan Ophthalmic Center. The *Brn3b*^*AP/AP*^ knockin mutant mice were previously generated and maintained in our laboratory (Gan et al., 1999). The Brn3b-GFP reporter mouse line was created by CRISPR/Cas9 gene editing, in which the *Brn3b* open reading frame was tethered to the GFP reporter gene by a P2A self-cleaving peptide sequence (Xiao et al., 2020). More related results about this line will be published elsewhere. The Glast-CreER mice (Heng et al., 2019) (stock number: 012586) were purchased from The Jackson Laboratory (Bar Harbor, ME, United States). The C57BL6 and CD1 mice were purchased from the Vital River Laboratories (Beijing, China). All genotypes were determined by PCR.

### Construction of Viral Plasmids and Adeno-Associated Virus Production and Injection

The control GFAP-GFP AAV vector containing an approximately 700 bp GFAP promoter (Kuzmanovic et al., 2003) was a gift from Bryan Roth (pAAV-GFAP-EGFP, Addgene plasmid #50473). The full-length open reading frames (ORFs) of murine Math5 and Brn3b alone or in combination via P2A or T2A were subcloned into this vector to construct desired AAV viral plasmids. By replacing GFP, we also used this vector as a backbone to construct the AAV plasmids expressing tdTomato or Cre. For construction of the FLEX Cre-Switch plasmid, the fusion ORF of Math5-P2A-Brn3b-T2A was amplified using a high fidelity DNA polymerase (Takara, R051S), and cloned into the *Kpn*I site of the CAG-FLEX-GFP vector (Addgene, #28304) by homologous recombination.

Adeno-associated virus production was performed as described previously with modification (Grieger et al., 2006). In brief, 20–24 h before transfection, AAV-293 cells (Sangon Biotech Co., Ltd., Shanghai, China) were cultured in 10 150-mm plates. For each plate, they were transfected with 6 μg of AAV vector DNA, 6 μg of AAV Rep/Cap plasmid DNA and 18 μg of adenovirus helper plasmid DNA using the Polyethylenimine (PEI) transfection method. After 60–72 h, the transfected cells were collected and resuspended in lysis buffer (150 mM NaCl, 20 mM Tris–HCl, pH8.0), then for three times frozen in dry ice/ethanol bath and thawed completely in 55°C water bath. The cell lysate was digested with Benzonase (50 U/ml) for 1 h at 37°C, and centrifuged to remove the cell debris. For purification, the virus-containing supernatant was applied to discontinuous iodixanol gradients followed by ultracentrifugation. The virus band was collected from the 40–60% interface using a syringe with a 21-gauge needle. The iodixanol solution was exchanged to 1x DPBS (Dulbecco’s phosphate-buffered saline) using the Amicon Ultra-15 centrifugal filter units from Millipore and the viruses were further concentrated by shrinking the volume. Virus titers were determined by qRT-PCR using linearized plasmid standards and primers against the ITR. AAVs were injected subretinally or intravitreally into adult mouse eyes using a microsyringe with a 33-gauge needle as described (Mo et al., 2004; Yao et al., 2018).

### Rabies-Virus *Trans*-Synaptic Tracing

For *trans-*synaptic tracing of the MG-derived RGCs→dLGN→V1 pathway, 0.6 μl of helper viruses containing a mixture of 0.2 μl each AAV9-hSyn-Cre, AAV9-EF1α-DIO-RVG, and AAV9-EF1α-DIO-BFP-T2A-TVA viruses (titer: 2–4 × 10^12^ particles/ml, BrainVTA, China) was injected into the dLGN of 2-month-old mice [AP (anterior-posterior: posterior to bregma): 2.2 mm; ML (midline to lateral): ±2.3 mm; DV (dorsoventral): 2.6 mm]. In addition, 2 μl of GFAP-Math5-Brn3b-tdTomato AAVs (titer: 2–4 × 10^12^ particles/ml) were injected subretinally into the eye. Three weeks later, 0.4 μl of ΔG-RABV-GFP rabies viruses (RV-ENVA-ΔG-EGFP, titer: 2 × 10^8^ particles/ml, BrainVTA, China) was injected into the V1 region (primary visual cortex) [AP (anterior to Lambda): 0.2–0.5 mm, ML: ±2.5 mm, DV: 0.15–0.5 mm]. Seven days after infection of rabies viruses, tissues were harvested and analyzed as described previously (Xiao et al., 2018).

### Tamoxifen Administration

100 mg of tamoxifen (T5648, Sigma) was first dissolved in 1 ml of ethanol and then 4 ml of corn oil was added to a final concentration of 20 mg/ml. Glast-CreER mice were administered with tamoxifen intraperitoneally at a daily dose of 200 mg/kg body weight for four consecutive days.

### Electrophysiology

Three weeks following infection of the eyes of adult wild-type and Brn3b-GFP reporter mice by GFAP-Math5-Brn3b-tdTomato AAVs, the retina was dissected out from the eyeball and its edge was removed to allow the tissue to lie flat. It was transferred to a recording chamber and bathed in external solution containing the Ames’ medium (Sigma-Aldrich). The chamber was mounted on a microscope equipped with a 40× water immersion objective. The cells and recording pipettes were viewed on a monitor that was coupled to a camera (Scientifica SciCam Pro, Canada). Oxygenated external solution was continuously perfused into the recording chamber at a flow rate of 1.5–2 ml/min. tdTomato-positive cells were identified with a mercury lamp (TH4-200, Olympus, Japan). Then, patch-clamp recordings were performed with a 700B amplifier (Molecular Devices, United States) and digitized at 10 kHz with a Digidata 1550B (Molecular Devices, United States). Alexa Fluor 488 hydrazide was used to label the patched cells. The action potentials of the cells were recorded under current clamp mode, with 6–9 MΩ resistance pipettes that were filled with an internal solution consisting of the following: 123 mM K-gluconate, 12 mM KCl, 10 mM HEPES, 0.2 mM EGTA, 4 mM Mg-ATP, 0.3 mM Na-GTP, 10 mM Na_2_-phosphocreatine, and 20 μg/ml glycogen (the pH value was adjusted to 7.25 with 0.5 M KOH). For current-clamp recording, we set the initial resting membrane potential (V-rest) to −55 mV using a small, constant holding current and applied current pulses with a step size of 20 pA to test the ability to generate action potentials. The cells were held at −55 mV under voltage clamp mode to record sPSCs, and the patch pipettes (6–9 MΩ) were filled with an internal solution containing: 40 mM CsCl, 90 mM K-gluconate, 1.8 mM NaCl, 1.7 mM MgCl_2_, 3.5 mM KCl, 0.05 mM EGTA, 10 mM HEPES, 2 mM Mg-ATP, 0.4 mM Na_2_-GTP, and 10 mM Na_2_-phosphocreatine (the pH value was adjusted to 7.25 with 0.1 M CsOH). To block responses mediated by ionotropic glutamate receptors, D-AP5 (50 μM) or CNQX (20 μM) was added to the external solution and perfused into the recording chamber.

For light response recordings, the dark-adapted mouse retina was isolated under far-red light and incubated in oxygenated Ames’ medium (Sigma, A1420) with constant bubbling (95% O_2_, 5% CO_2_) at room temperature. Four sections were made to flat-mount the retina with RGCs facing up in a superfusion chamber on the stage of a custom-built upright fluorescence microscope. The recording chamber was perfused with Ames solution at 31–33°C and RGC bodies were visualized and recognized using upright IR light and green fluorescence. Light-evoked spikes of labeled RGCs were obtained with whole-cell current-clamp recording (Heka patch system) using patch pipettes, which had an impedance of 3–4 MΩ and were filled with high potassium internal solution (116 mM K^+^ glucose, 12 mM KCl, 10 mM HEPES, 10 mM EGTA, 4 mM Mg-ATP, 0.3 mM Na-GTP, 0.3 mM CaCl_2_, 0.5 mM MgCl_2_). Light stimuli were delivered from a modified mercury bulb (Olympus) with band-pass filtering (530–550 nm, green), and focused onto the RGC side of the retina through a 40X water immersion objective. Intensities of green and blue light were equal and measured (log_10_ 7 photons/s/μm^2^) using the calibrated photometer (Thorlabs, PM100D, S170C). Data were analyzed using MATLAB (MathWorks).

### Optic Nerve Crush Injury

Mice were anesthetized by intraperitoneal injection of 4% chloral hydrate and one drop of 0.4% oxybuprocaine hydrochloride was administered for local anesthesia. ONC was performed as described (Li et al., 1999). Briefly, a small incision was made with scissors in the conjunctiva of the left eye located at the 3–9 o’clock of eyeball. The exposed optic nerve was grasped approximately 1 mm from the eyeball with forceps for 10 s. It was then released to allow the eyeball to rotate back into place. Three days following ONC, the left eye of each animal was infected with AAVs by subretinal injection.

### Visual Evoked Potential Test

Two to six weeks after AAV injection, the ONC and *Brn3b*^*AP/AP*^ mice were dark-adapted overnight, prior to being prepared for the experiments. They were anesthetized by intraperitoneal injection of 4% chloral hydrate and their pupils were dilated with a drop of tropicamide. One drop of 0.4% oxybuprocaine hydrochloride was administered for local anesthesia of the cornea.

During VEP recordings which were carried out using the Celeris ERG system (Diagnosys LLC, Lowell, MA, United States), the animals were placed on a heated platform to keep warm. Needle electrodes placed subcutaneously at the base of the tail and at the snout served as ground and reference electrodes, respectively. The active electrode was inserted subcutaneously at the midline at the back of the head. Two contact-lens light-emitting diodes (LEDs) were placed over the two eyes of the animal to serve as light stimulators. Each eye was separately exposed to white light flashes of 0.05 cd.s/m^2^, swept 100 times per trial. For each mouse, we performed five trials. Analyses were performed using GraphPad Prism 8.

### Immunohistochemistry

Immunostaining of retinal sections, retinal flat-mounts and optic nerves were carried out as previously described (Xiang et al., 1995; Li et al., 2004). Mouse brain tissues were immunostained as free floating sections also as previously described (Xiao et al., 2018). In brief, for section labeling, retinas were fixed in 4% paraformaldehyde (PFA) in PBS for 30 min at 4°C and sectioned at 14 μm. Sample sections were washed three times with 0.1% Tween in PBS (PBST) for 5 min each before being incubated in 5% normal donkey serum in PBST for 1 h at room temperature (RT). Then primary antibodies in 2% normal donkey serum in PBST were added for overnight incubation at 4°C. After washing with PBST, the sections were incubated with secondary antibodies and DAPI in PBST for 1 h at RT. Images were captured by a laser scanning confocal microscope (Carl Zeiss, LSM700).

The following primary antibodies were used: GFP (Abcam, ab6673, 1:2000), GFP (MBL, 598, 1:2000), RFP (Rockland antibodies and assays, 40657, 1:1000), tdTomato (Kerafast, EST203, 1:2000), Rbpms (Novus, NBP2-20112, 1:1000), Brn3a (Millipore, MAB1585, 1:500), Brn3b (Santa Cruz, SC-390780, 1:1000), Brn3c (Proteintech, 21509-1-AP, 1:1000), Sox9 (Millipore, ab5535, 1:1000), Sox9 (Abnova, H00006662-M02, 1:2000), Tfap2a and b (Abcam, ab11828, 1:1000), Pax2 (R&D Systems, AF3364, 1:1000), GFAP (DAKO, Z0334, 1:2000), S100β (Abcam, ab52642, 1:2000), Tuj1 (Covance, MMS-435P, 1:1000), melanopsin (Thermo Fisher Scientific, PA1-781, 1:100), Eomes (Abcam, ab23345, 1:2000), peripherin (Millipore, ab1530, 1:1000), Foxp2 (Abcam, ab16046, 1:4000), and Satb2 (Abcam, ab51501, 1:4000).

### Single-Cell RNA Sequencing Analysis

Single-cell RNA sequencing (scRNA)-seq analysis was carried out as previously described (Xiao et al., 2020). In brief, 5.5 days after infection of adult mouse retinas with GFAP-Math5-Brn3b-GFP AAVs, two retinas were quickly dissected and dissociated using papain with DNase I at 37°C for 5 min. Then isometric amount of DPBS containing 10% FBS was added and retinas were triturated by soft pipetting for dissociation. The dissociated cells were filtered using a 40-μm cell strainer. Filtered cells were centrifuged and resuspended with 2 ml DPBS containing 5% FBS. GFP^+^ retinal cells were then enriched by fluorescence-activated cell sorting (FACS) using the FACSAria Fusion cell sorter (BD Biosciences). Single-cell libraries were generated from the enriched GFP+ cells and sequenced on the Illumina X Ten platform (Berry Genomics, China). Further analyses were performed using Seurat and Monocle (Xiao et al., 2020).

### EdU Labeling and Detection

Following infection of adult mouse retinas with GFAP-Math5-Brn3b-GFP AAVs, 2 μl of EdU solution (1 mg/ml) was injected into the vitreous chamber of each eye at different time points. For sample collection, under deep anesthesia induced with intraperitoneal injection of chloral hydrate (4.5 μg/g body weight), mice were intracardially perfused with cold PBS for 5 min, followed by 4% cold PFA in PBS for 15 min. The eyeballs were isolated and post-fixed in 4% PFA for 1 h at 4°C. The retinas were dissected out and the vitreous was completely removed. They were shaped into a “petal” by 4–5 radial incisions, flattened in a 48-well plate, and permeabilized with 0.3% Triton-100 in PBST for 15 min at RT. After incubation in 10% normal donkey serum in PBST for 2 h at RT, the retinas were incubated in primary antibodies against Sox9 and GFP diluted in 2% normal donkey serum in PBST for 2 days at 4°C. Retinas were washed with PBST and incubated with secondary antibodies for 2 h. After three washes with PBS, the retinas were stained for EdU with the Click-iT EdU Kit (Invitrogen). Images were captured by a laser scanning confocal microscope (Carl Zeiss, LSM700).

### Transmission Electron Microscopy

One month following infection of adult mouse retinas with GFAP-Math5-Brn3b-tdTomato AAVs or GFAP-tdTomato AAVs, electron microscopy of corresponding optic nerves was performed as previously described (Gan et al., 1996; Liu et al., 2020).

### Statistics

Statistical analysis was performed using the GraphPad Prism 8 and Microsoft Excel computer programs. The results are expressed as mean ± SD for experiments conducted at least in triplicates. Unpaired two-tailed Student’s *t*-test and one-way or two-way ANOVA with Bonferroni’s correction were used to test for significance, and a value of *P* < 0.05 was considered statistically significant.

## Data Availability Statement

The datasets presented in this study can be found in online repositories. The names of the repository/repositories and accession number(s) can be found below: NCBI (accession: PRJNA648671).

## Ethics Statement

The animal study was reviewed and approved by the Institutional Animal Care and Use Committee (IACUC), Zhongshan Ophthalmic Center, Sun Yat-sen University.

## Author Contributions

DX, KJ, SL, YL, and MX conceived and designed the research. DX, KJ, SQ, QL, WH, HC, BG, ZX, XT, FL, QX, MX, and JS performed the experiments and analyzed the data. DX, KJ, and MX interpreted the data and wrote the manuscript. All authors contributed to critical reading of the manuscript.

## Conflict of Interest

The authors declare that the research was conducted in the absence of any commercial or financial relationships that could be construed as a potential conflict of interest.

## Publisher’s Note

All claims expressed in this article are solely those of the authors and do not necessarily represent those of their affiliated organizations, or those of the publisher, the editors and the reviewers. Any product that may be evaluated in this article, or claim that may be made by its manufacturer, is not guaranteed or endorsed by the publisher.
