# Supplementary material for: In vivo Regeneration of Ganglion Cells for Vision Restoration in Mammalian Retinas
Source: Front Cell Dev Biol. 2021 Oct 4;9:755544. doi: 10.3389/fcell.2021.755544 (PMC8520940; doi:10.3389/fcell.2021.755544)
Supplement: Supplementary file 1 [file Data_Sheet_1.PDF]

# In Vivo Regeneration of Ganglion Cells for Vision Restoration in Mammalian Retinas

**Dongchang Xiao[1,#], Kangxin Jin[1,#], Suo Qiu[1], Qiannan Lei[1], Wanjing Huang[1], Haiqiao Chen[1], Jing Su[1], Qiang Xu[1], Zihui Xu[1], Bin Gou[1], Xiaoxiu Tie[1], Feng Liu[1], Sheng Liu[1,*], Yizhi Liu[1,*], and Mengqing Xiang[1,2,*]**

## Supplementary Figures

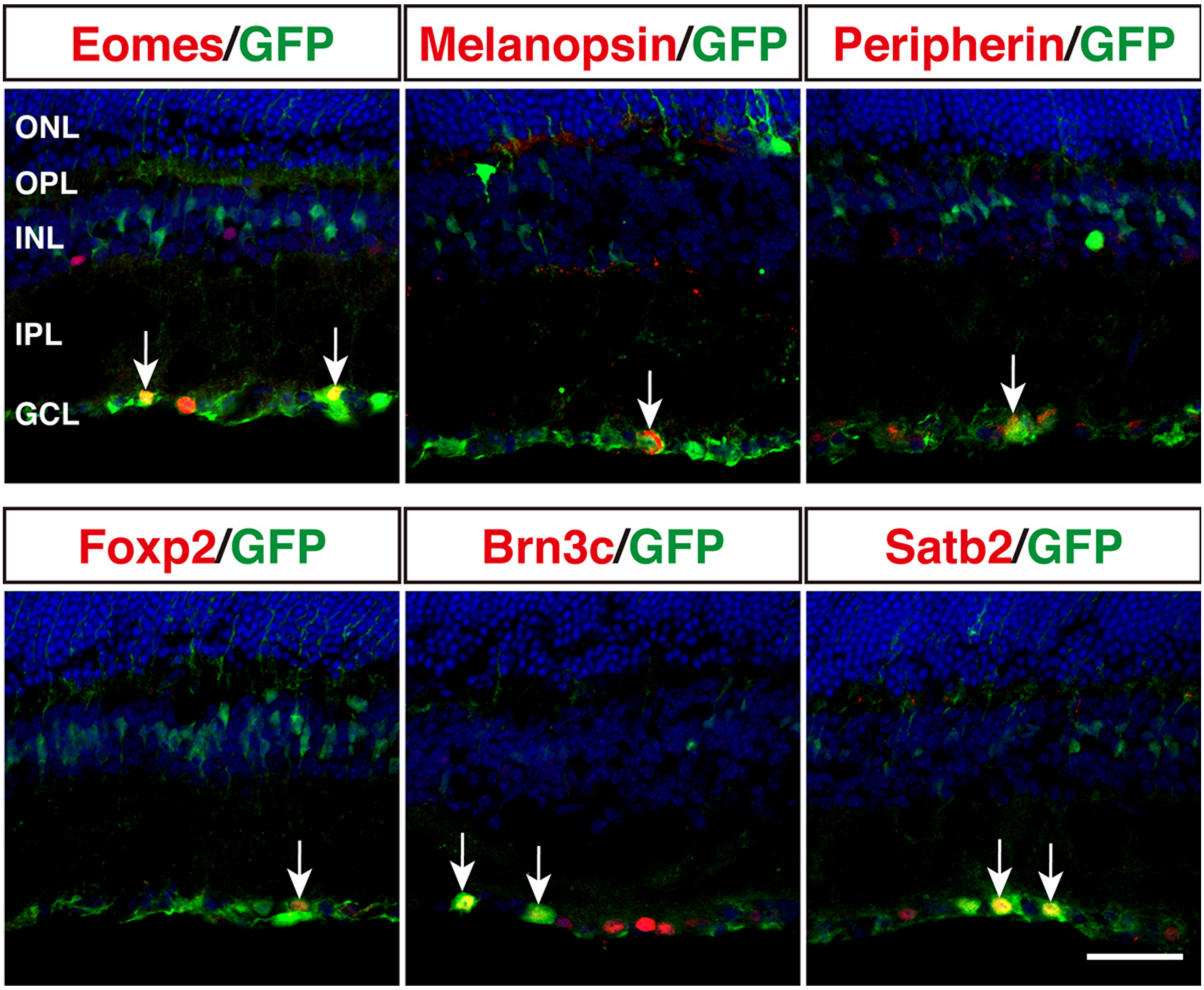


**Figure S1. RGC subtypes reprogrammed from MG by Math5 and Brn3b.** Three weeks after infection with GFAP-Math5-Brn3b-GFP AAVs, sections from infected retinas were double-immunolabeled with the indicated antibodies and counterstained with nuclear DAPI. Arrows point to colabeled cells. Abbreviations: GCL, ganglion cell layer; INL, inner nuclear layer; IPL, inner plexiform layer; ONL, outer nuclear layer; OPL, outer plexiform layer. Scale bar: 40 μm.

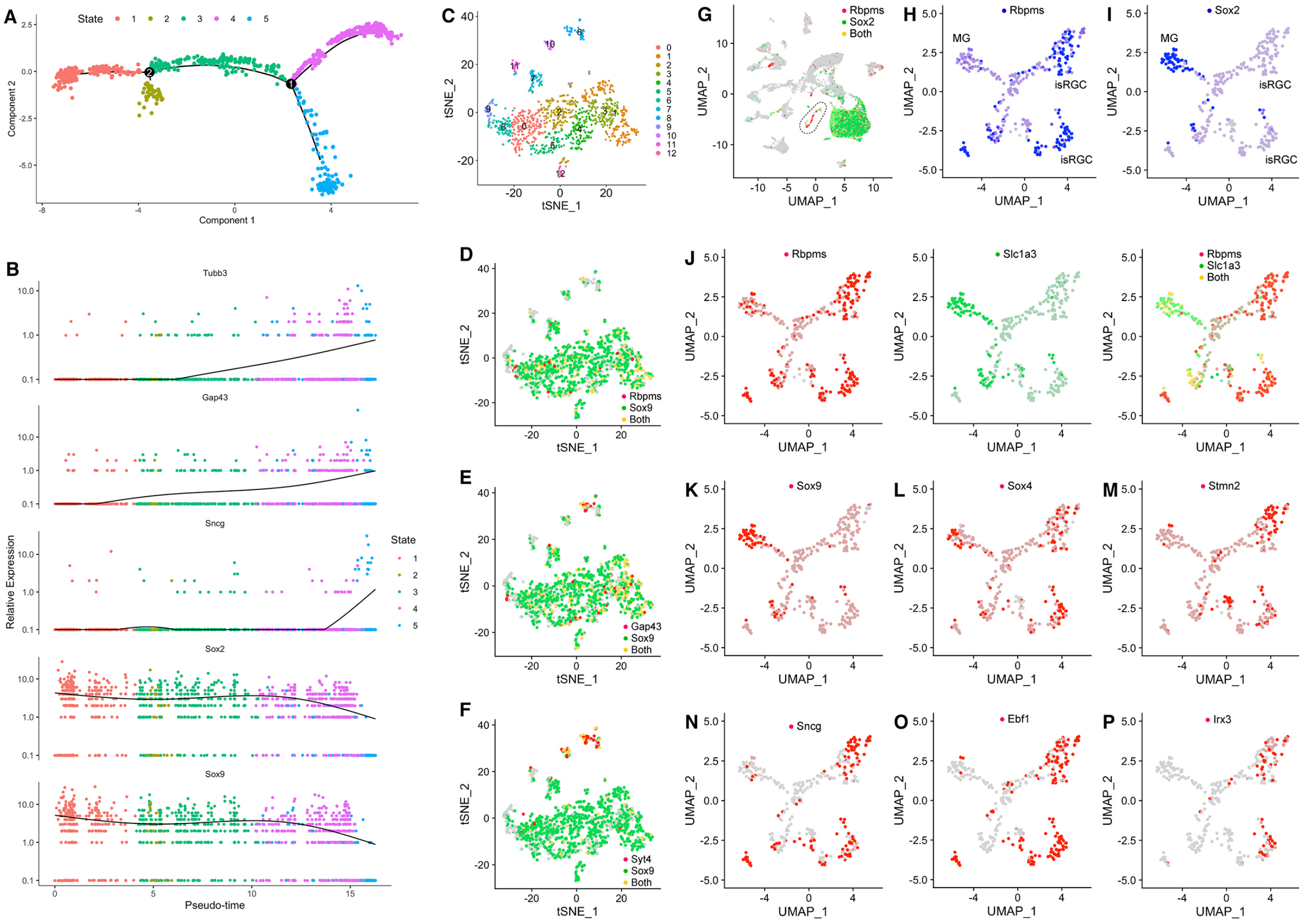

**Figure S2. Intermediate stages of MG transdifferentiation detected by single cell transcriptome profiling.** (**A**) At 5.5 days after infection with GFAP-Math5-Brn3b-GFP AAVs, GFP+ cells were enriched and subjected to scRNA-seq. A pseudotime trajectory of the sequenced GFP+ cells was constructed using Monocle. Indicated in color are the five presumptive states and corresponding cell population. (**B**) Relative expression levels of *Tubb3, Gap43, Sncg, Sox2,* and *Sox9* in pseudotime. (**C**) t-SNE plot of cell clusters generated from the sequenced GFP+ cells. (**D-F**) t-SNE plots colored by expression of the indicated RGC and MG marker genes. (**G**) UMAP plot of all sequenced cells colored by expression of Rbpms and Sox2. The related cell populations circled by the dashed line are re-analyzed by UMAP in panels H-P. (**H-P**) UMAP plots of the highlighted cell populations in panel G, colored by expression of the indicated RGC and MG marker genes. Abbreviation for isRGC: intermediate stage RGC.

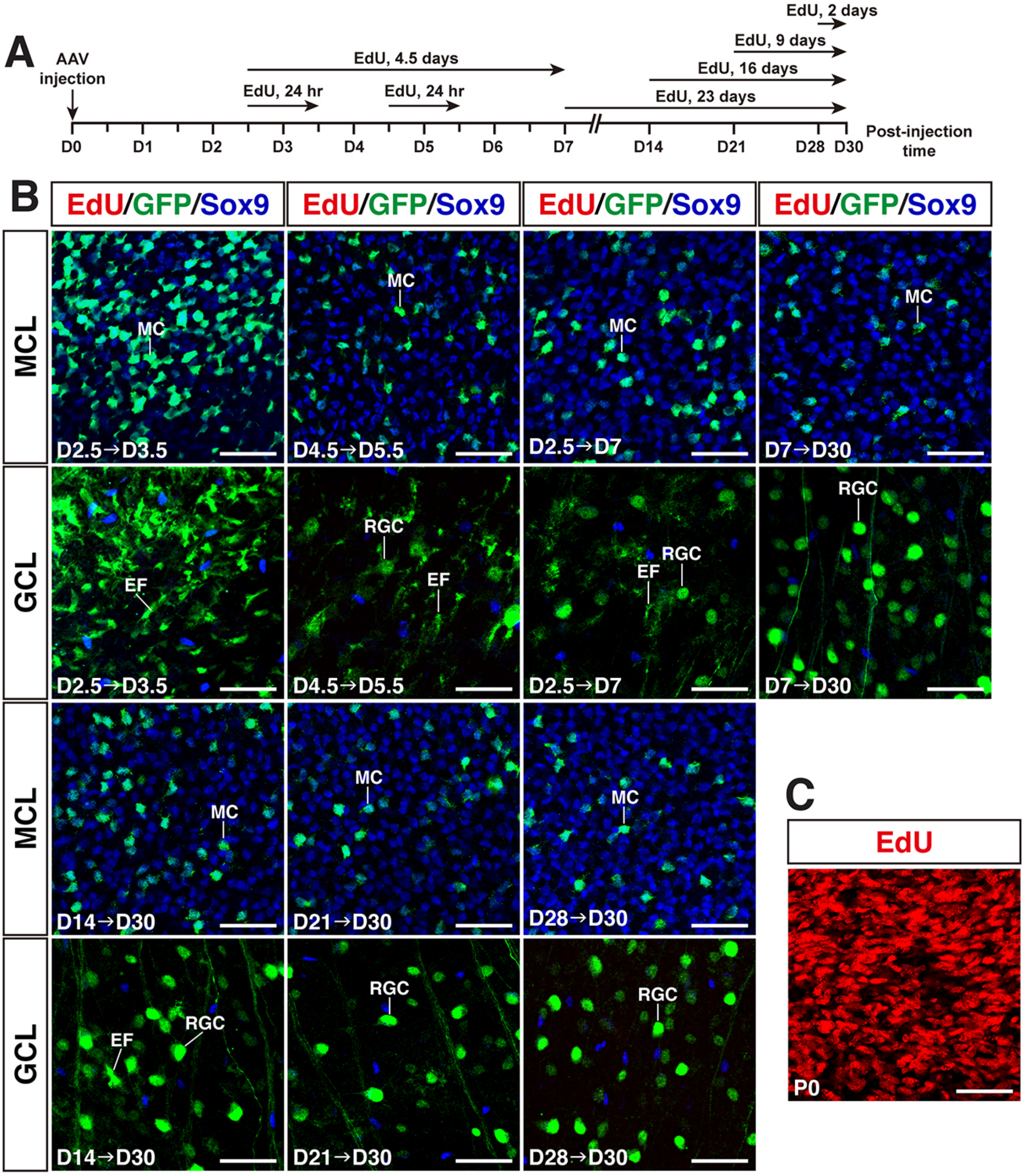


**Figure S3. Math5 and Brn3b together do not trigger proliferation of mature MG.** (**A**) Schematic of EdU labeling schedule following infection of adult mouse retinas with GFAP-Math5-Brn3b-GFP AAVs. (**B**) Flat-mounts of adult mouse retinas infected with AAVs and pulse-labeled by EdU were fluorescently stained for EdU and immunostained for both GFP and Sox9. The time (D, day) of EdU injection and retina collection is indicated on each panel. The confocal images are focused on the Müller cell layer (MCL) or ganglion cell layer (GCL). There are no EdU-positive cells present. (**C**) Flat-mounts of P0 mouse retinas pulse-labeled by EdU were fluorescently stained for EdU. There are numerous EdU-positive cells present. Abbreviations: EF, MG endfoot; MC, Müller cell; RGC, retinal ganglion cell. Scale bars: 40 μm (B,C).

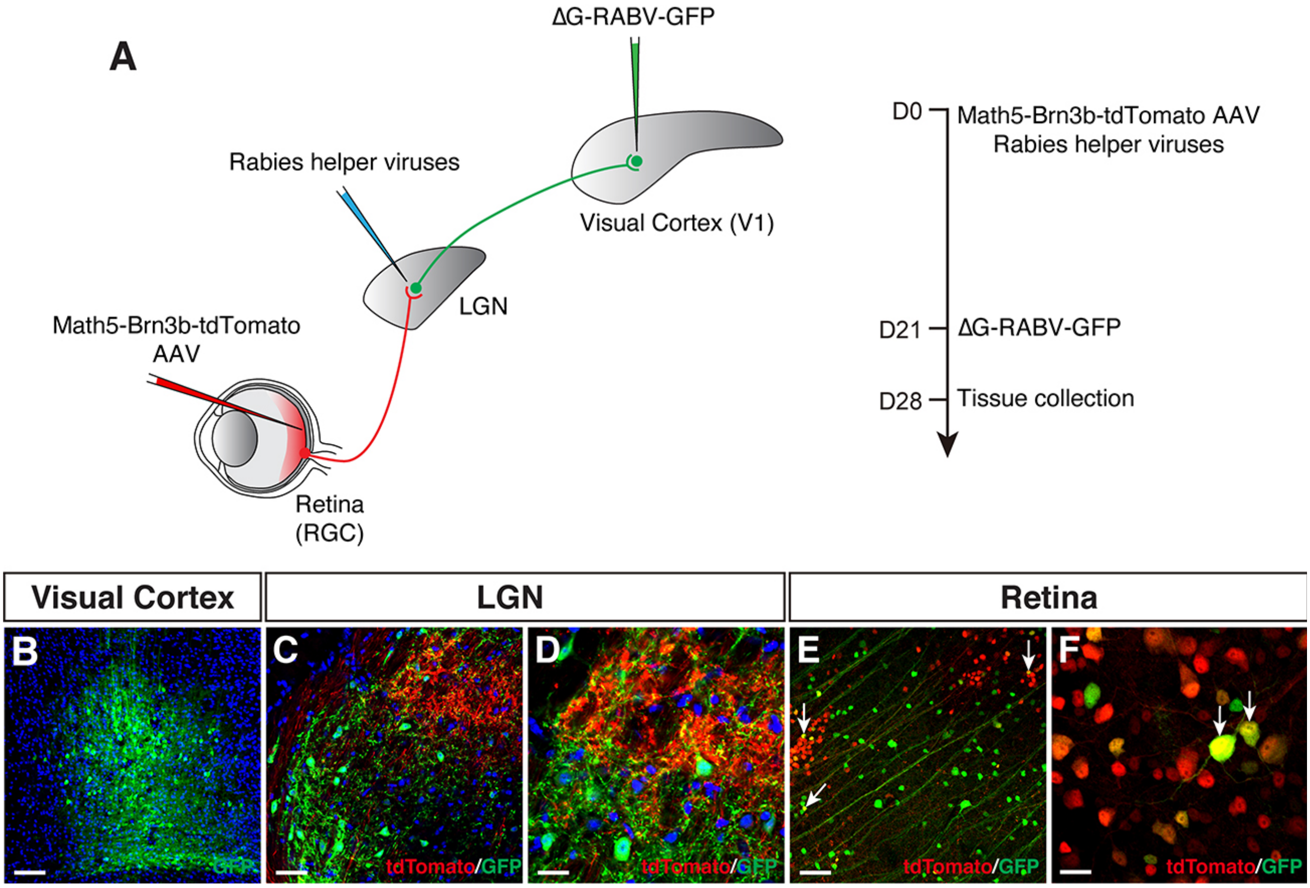


**Figure S4. Rabies-virus trans-synaptic network tracing of MG-derived RGCs.** (**A**) Experimental design and timing (D, day) of virus injection and tissue collection. (**B-F**) Sections from injected primary visual cortex (B) and lateral geniculate nucleus (LGN) (C,D) or flat-mount retinas (E,F) were immunolabeled with the indicated antibodies. Sections were also counterstained with DAPI. There are visible contacts between tdTomato-immunoreactive RGC axons and GFP-labeled LGN cells/processes (C,D), and some RGCs (indicated by arrows in E,F) immunoreactive for both tdTomato and GFP in the retina (E,F). Scale bar: 80 μm (B), 40 μm (C,E), 20 μm (D,F).

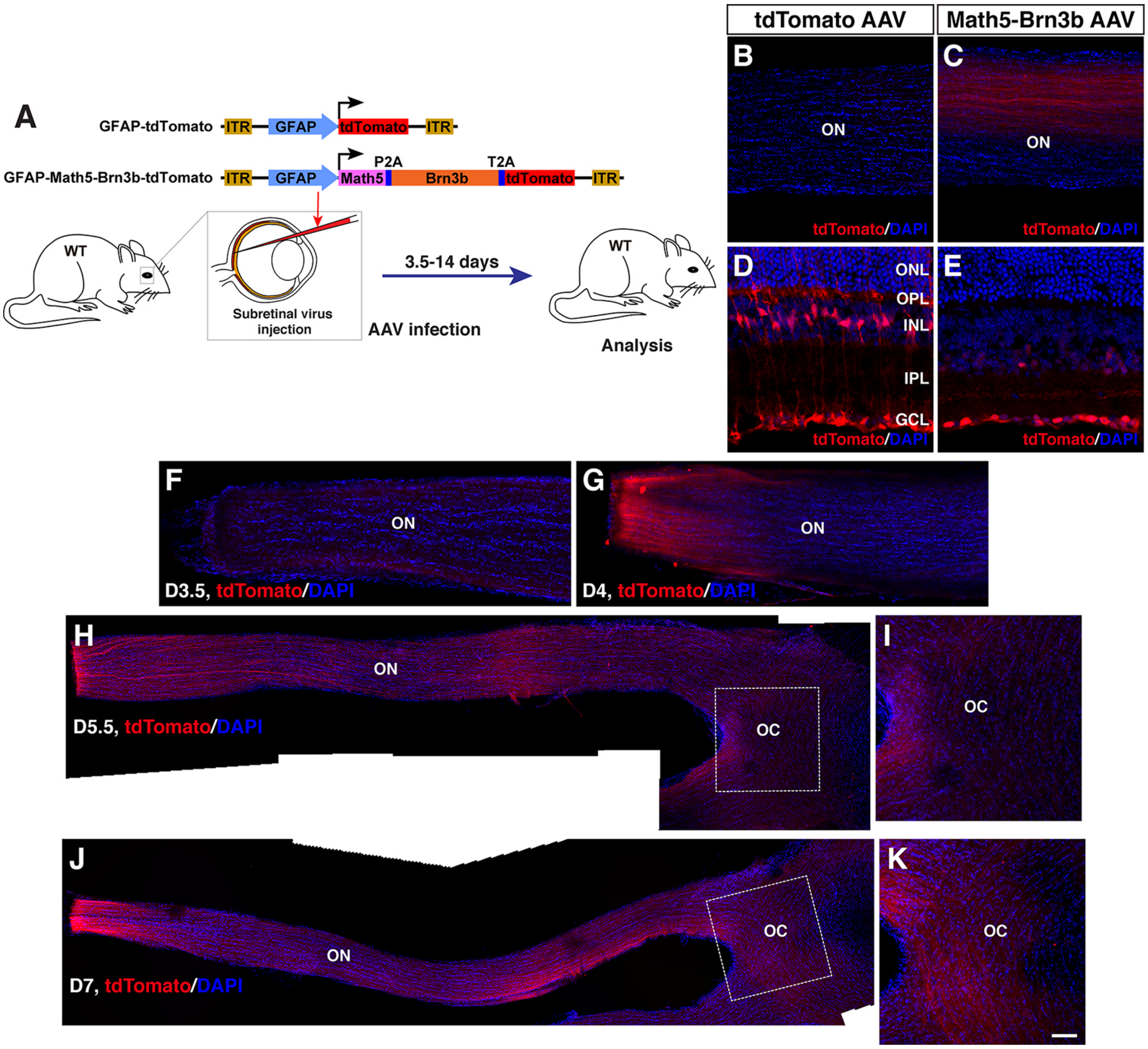


**Figure S5. Temporal progression of MG-derived RGC projections.** (**A**) Schematic of the AAV constructs and infection procedure in adult wild-type (WT) mice. (**B-E**) Two weeks after viral infection, the optic nerves of mice treated with GFAP-Math5-Brn3b-tdTomato or GFAP-tdTomato AAVs were visualized for tdTomato fluorescence without antibody labeling (B, C). Retinal sections from the corresponding mice were immunostained with an anti-tdTomato antibody (D, E). They were all counterstained with nuclear DAPI. (**F-K**) At 3.5 (D3.5), 4, 5.5 and 7 days after viral infection, the optic nerves, optic chiasms and optic tracts of mice treated with GFAP-Math5-Brn3b-tdTomato AAVs were immunolabeled with an anti-tdTomato antibody and counterstained with nuclear DAPI. The outlined regions in (H) and (J) are shown at a higher magnification in (I) and (K), respectively. Images in (H, J) are stitched from serial micrographs. Abbreviations: GCL, ganglion cell layer; INL, inner nuclear layer; IPL, inner plexiform layer; OC, optic chiasm; ON, optic nerve; ONL, outer nuclear layer; OPL, outer plexiform layer. Scale bar: 160 μm (H,J), 80 μm (B,C,F,G,I,K), 20 μm (D,E).

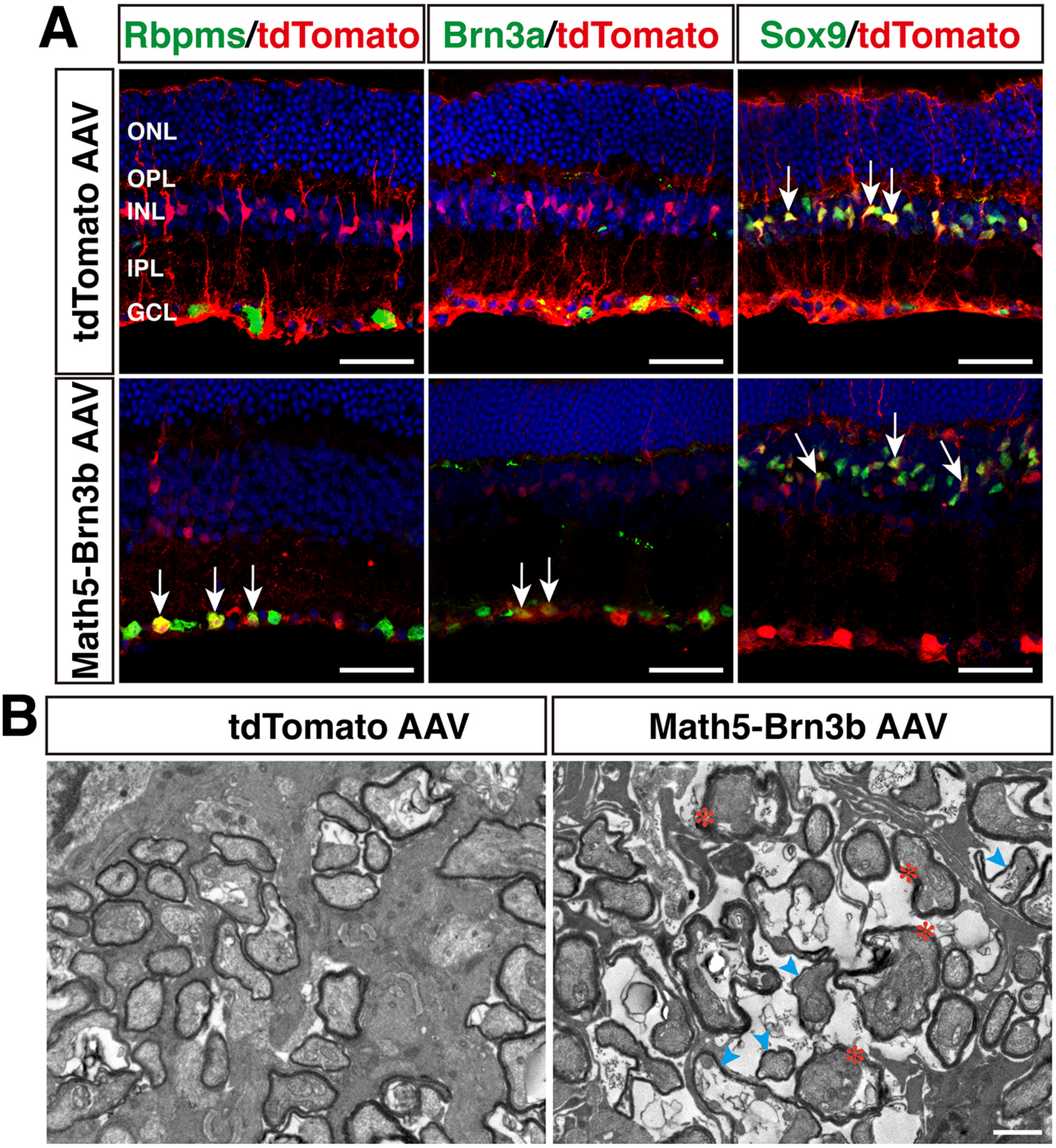


**Figure S6. RGC regeneration in the *Brn3b*$^{AP/AP}$ retina.** (**A**) Sections from *Brn3b*$^{AP/AP}$ retinas infected with GFAP-tdTomato or GFAP-Math5-Brn3b-tdTomato AAVs at one month of age were double-immunostained with antibodies against tdTomato and Rbpms, Brn3a or Sox9. They were also counterstained with nuclear DAPI. Arrows point to representative colabeled cells. Compared to the control, Math5 and Brn3b together increased RGCs co-immunoreactive for both tdTomato and Rbpms or Brn3a. (**B**) Electron micrographs of cross sections of the optic nerves from *Brn3b*$^{AP/AP}$ mice infected with GFAP-tdTomato or GFAP-Math5-Brn3b-tdTomato AAVs. The asterisks indicate axons with thick myelin sheath and the arrowheads indicate those with thin myelin sheath. Abbreviations: GCL, ganglion cell layer; INL, inner nuclear layer; IPL, inner plexiform layer; ONL, outer nuclear layer; OPL, outer plexiform layer. Scale bars: 40 μm (A), 10 μm (B).

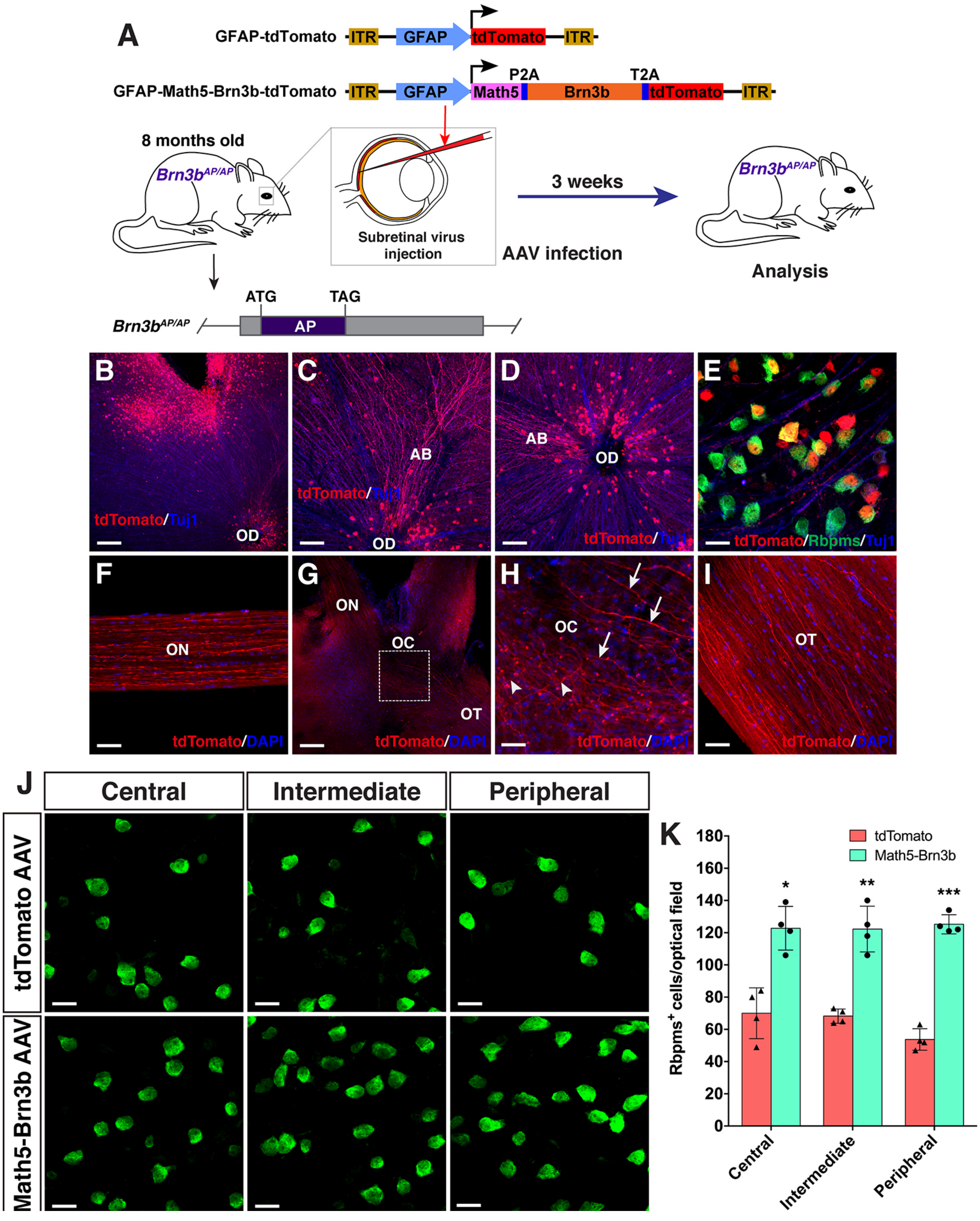


**Figure S7. RGC regeneration in aged *Brn3b*$^{AP/AP}$ mice.** (**A**) Schematic of the AAV constructs and infection procedure to regenerate RGCs in *Brn3b*$^{AP/AP}$ mice at 8 months of age. (**B-E**) Flat-mounts of *Brn3b*$^{AP/AP}$ retinas treated with GFAP-Math5-Brn3b-tdTomato AAVs were double-immunolabeled with anti-tdTomato and anti-Tuj1 antibodies (B-D), or triple-immunostained with antibodies against tdTomato, Rbpms and Tuj1 (E). (**F**) An optic nerve

from *Brn3b*$^{AP/AP}$ mice treated with GFAP-Math5-Brn3b-tdTomato AAVs was immunoreactive for tdTomato. (**G-I**) The optic nerves, optic chiasms and optic tracts from *Brn3b*$^{AP/AP}$ mice treated with GFAP-Math5-Brn3b-tdTomato AAVs were immunoreactive for tdTomato. Shown in (H) is a higher magnification view of the region outlined in (G). Arrows in (H) point to axons crossing the midline of the optic chiasm (contralateral projection) while arrowheads indicate non-crossing axons (ipsilateral projection). (**J**) Flat-mounts of central, intermediate and peripheral *Brn3b*$^{AP/AP}$ retinas treated with GFAP-tdTomato or GFAP-Math5-Brn3b-tdTomato AAVs were immunostained with an anti-Rbpms antibody. (**K**) Quantification of Rbpms+ cells in central, intermediate and peripheral *Brn3b*$^{AP/AP}$ retinas treated with GFAP-tdTomato or GFAP-Math5-Brn3b-tdTomato AAVs. Data are presented as mean ± SD (n=4). Asterisks indicate significance in unpaired two-tailed Student's t-test: *p<0.005, **p<0.0005, ***p<0.0001. Abbreviations: AB, axon bundle; OC, optic chiasm; OD, optic disc; ON, optic nerve; OT, optic tract. Scale bars: 160 μm (B, G), 80 μm (C, D), 40 μm (F, H, I), 20 μm (E, J).

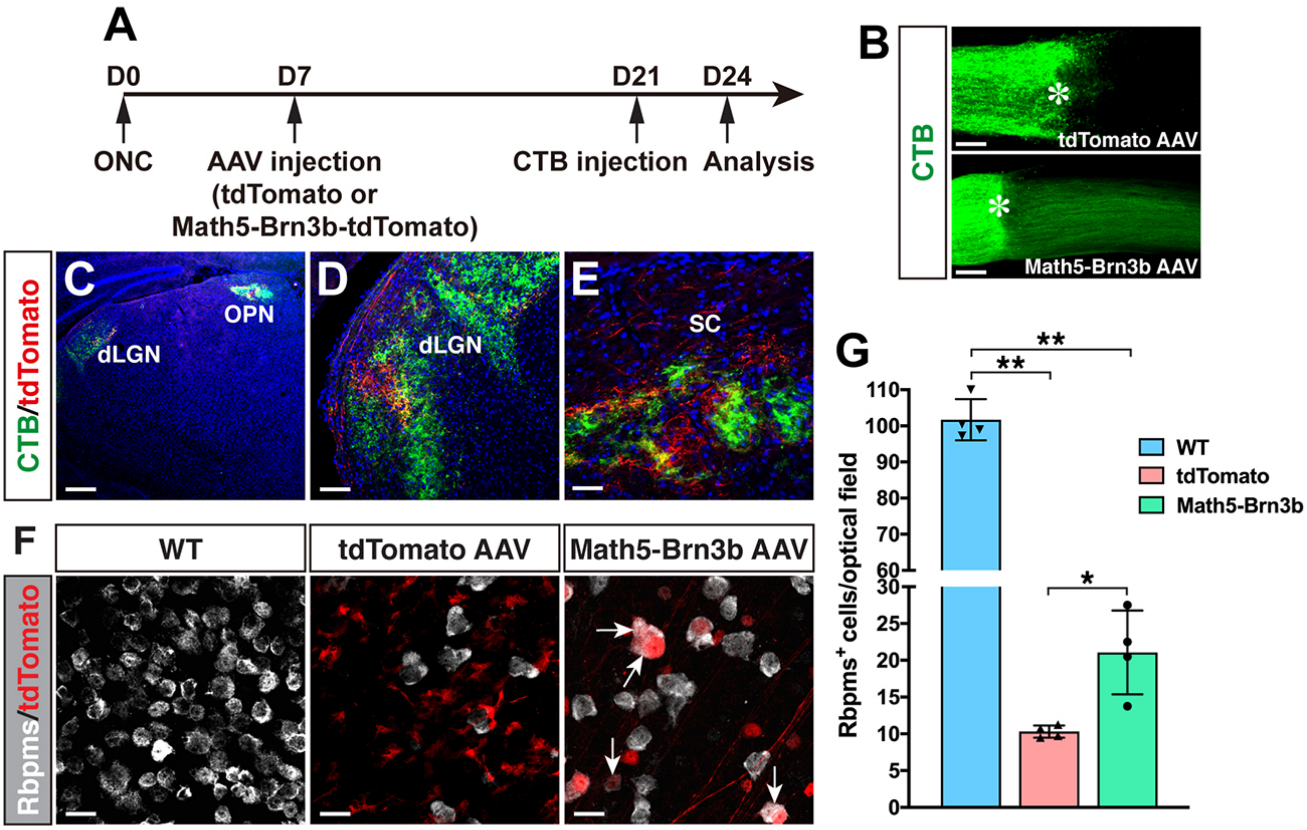


**Figure S8. Projections of MG-derived RGCs in ONC mouse models.** (**A**) Schematic of the experimental schedule. (**B**) Axons were labeled by CTB in optic nerves of ONC mouse models treated with GFAP-Math5-Brn3b-tdTomato AAVs or GFAP-tdTomato AAVs. The asterisk denotes the injury site and numerous axons project beyond this site in the treated optic nerve. (**C-E**) In ONC mice treated with GFAP-Math5-Brn3b-tdTomato AAVs, brain areas innervated by MG-derived RGCs were visualized in sections by CTB labeling and tdTomato immunostaining. The brain sections were also counterstained with nuclear DAPI. (**F**) Flat-mounts of wild-type (WT, without any treatment) retinas and ONC mouse retinas treated with GFAP-tdTomato or GFAP-Math5-Brn3b-tdTomato AAVs were double-immunostained with anti-tdTomato and anti-Rbpms antibodies. Arrows point to representative colabeled cells. (**G**) Quantification of Rbpms+ cells in the intermediate region of WT retinas and ONC retinas treated with GFAP-tdTomato or GFAP-Math5-Brn3b-tdTomato AAVs. Data are presented as mean ± SD (n=4). Asterisks indicate significance in one-way ANOVA test with Bonferroni's correction: *p<0.05, **p<0.001. Abbreviations: CTB, cholera toxin B fragment; dLGN, dorsal lateral geniculate nucleus; ONC, optic nerve crush; OPN, olivary pretectal nucleus; SC, superior colliculus;. Scale bar: 320 μm (C), 80 μm (B,D), 40 μm (E), 20 μm (F).

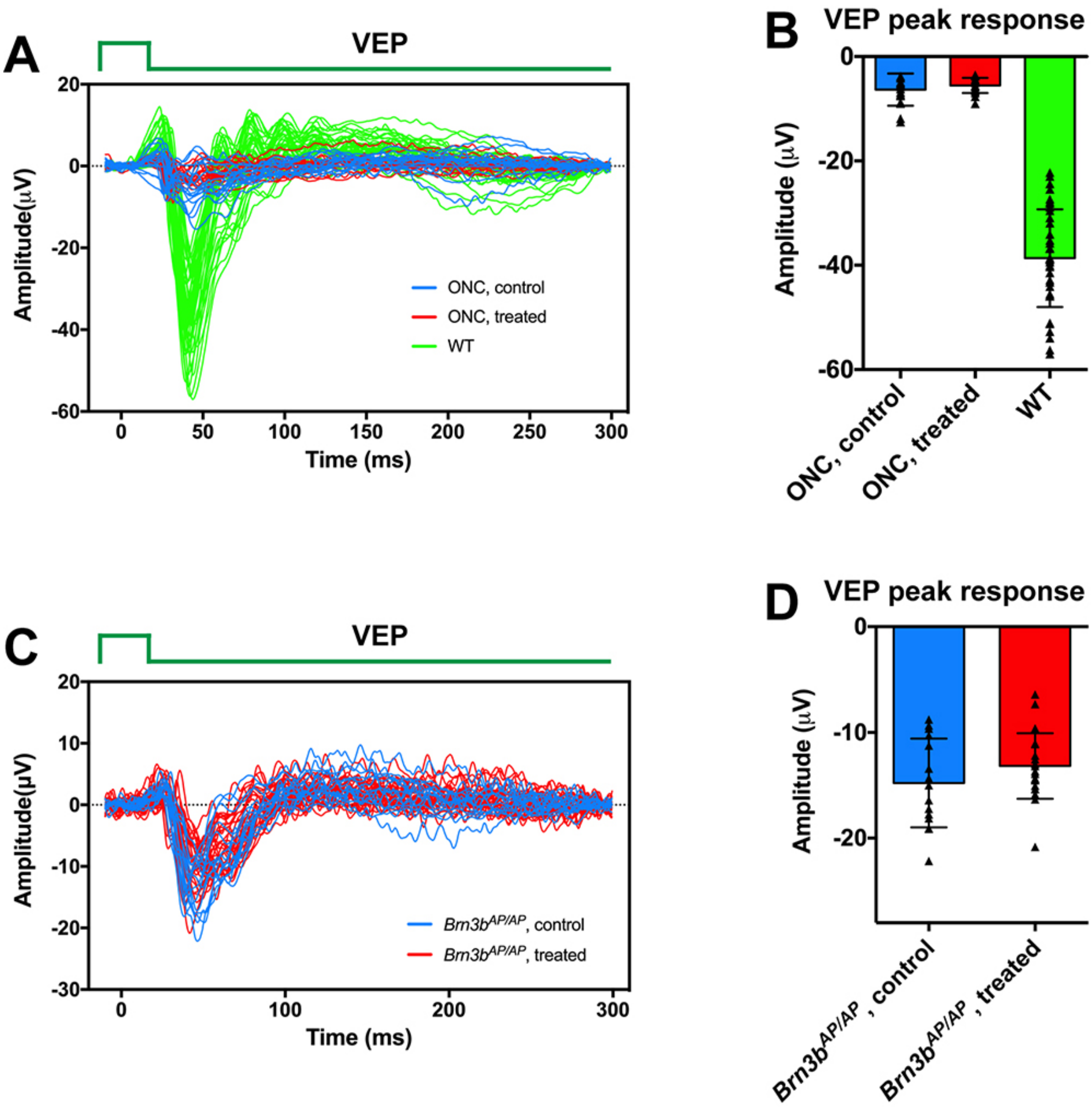


**Figure S9. Visual evoked responses in mouse models of RGC loss at two weeks after AAV treatment.** (**A, B**) Visual evoked responses (VEPs) to a flash light in the visual cortex of ONC mouse models in right eyes (WT, without optic nerve crush, n=13) and in left eyes (optic nerve crushed) treated with GFAP-Math5-Brn3b-GFP AAVs (ONC, treated, n=7) or GFAP-GFP AAVs (ONC, control, n=6). Shown in (A) are responses from all trials and five trials were performed for each eye. Shown in (B) are amplitudes of the VEP response peaks for control, treated and WT eye groups. Points represent single trials. Data are presented as mean ± SD. (**C, D**) VEPs to a flash light in the visual cortex of *Brn3b*$^{AP/AP}$ mice treated with GFAP-Math5-Brn3b-tdTomato AAVs (*Brn3b*$^{AP/AP}$, treated, n=5 eyes) or GFAP-tdTomato AAVs (*Brn3b*$^{AP/AP}$, control, n=3 eyes). Shown in (C) are responses from all trials and five trials were performed for each eye. Shown in (D) are amplitudes of the positive VEP response peaks for control and treated eye groups. Points represent single trials. Data are presented as mean ± SD.
